# Discovery and characterization of potent And‐1 inhibitors for cancer treatment

**DOI:** 10.1002/ctm2.627

**Published:** 2021-12-19

**Authors:** Jing Li, Yi Zhang, Jing Sun, Leyuan Chen, Wenfeng Gou, Chi‐Wei Chen, Yuan Zhou, Zhuqing Li, David W. Chan, Ruili Huang, Huadong Pei, Wei Zheng, Yiliang Li, Menghang Xia, Wenge Zhu

**Affiliations:** ^1^ Department of Biochemistry and Molecular Medicine The George Washington University School of Medicine and Health Sciences Washington District of Columbia USA; ^2^ GW Cancer Center The George Washington University Washington District of Columbia USA; ^3^ Tianjin Key Laboratory of Radiation Medicine and Molecular Nuclear Medicine, Institute of Radiation Medicine Peking Union Medical College & Chinese Academy of Medical Sciences Tianjin China; ^4^ Department of Obstetrics and Gynecology, LKS Faculty of Medicine The University of Hong Kong Hong, China; ^5^ Division of Preclinical Innovation, National Center for Advancing Translational Sciences National Institutes of Health Bethesda Maryland USA

**Keywords:** And‐1, cancer treatment, DNA replication, high throughput screen, small molecular

## Abstract

Acidic nucleoplasmic DNA‐binding protein 1 (And‐1), an important factor for deoxyribonucleic acid (DNA) replication and repair, is overexpressed in many types of cancer but not in normal tissues. Although multiple independent studies have elucidated And‐1 as a promising target gene for cancer therapy, an And‐1 inhibitor has yet to be identified. Using an And‐1 luciferase reporter assay to screen the Library of Pharmacologically Active Compounds (LOPAC) in a high throughput screening (HTS) platform, and then further screen the compound analog collection, we identified two potent And‐1 inhibitors, bazedoxifene acetate (BZA) and an uncharacterized compound [(*E*)‐5‐(3,4‐dichlorostyryl)benzo[*c*][1,2]oxaborol‐1(3*H*)‐ol] (CH3), which specifically inhibit And‐1 by promoting its degradation. Specifically, through direct interaction with And‐1 WD40 domain, CH3 interrupts the polymerization of And‐1. Depolymerization of And‐1 promotes its interaction with E3 ligase Cullin 4B (CUL4B), resulting in its ubiquitination and subsequent degradation. Furthermore, CH3 suppresses the growth of a broad range of cancers. Moreover, And‐1 inhibitors re‐sensitize platinum‐resistant ovarian cancer cells to platinum drugs in vitro and in vivo. Since BZA is an FDA approved drug, we expect a clinical trial of BZA‐mediated cancer therapy in the near future. Taken together, our findings suggest that targeting And‐1 by its inhibitors is a potential broad‐spectrum anti‐cancer chemotherapy regimen.

## Graphical abstract

Although multiple independent studies have elucidated And‐1 as a promising target gene for cancer therapy, And‐1 inhibitor has yet to be identified. Using an And‐1 luciferase reporter assay to screen Library of Pharmacologically Active Compounds (LOPAC) in a high throughput screening (HTS) platform, and then further screen the compound analog collection, we identified two potent And‐1 inhibitors, which induce And‐1 degradation.

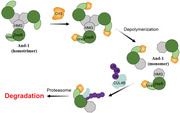

## 1 INTRODUCTION

Deoxyribonucleic acid (DNA) replication is a complex biological process that ensures accurate duplication of the genome. Inhibition of DNA replication provokes replication fork stalling, leading to genomic instability and apoptosis. Therefore, for decades inhibition of replication machinery has been regarded as a powerful anti‐cancer strategy, and many chemotherapy drugs (e.g., fluorouracil, bleomycin, thiotepa, cisplatin, etc.) have been approved to treat cancers because of their inhibition on DNA replication.^1^, ^2^, ^3^ Cisplatin is one of the most employed chemotherapy drugs that exhibit intense clinical activity against a wide array of solid neoplasms, including testicular, bladder, colon, ovarian, etc.^4^, ^5^ Cisplatin kills cancer cells by inducing both interstrand DNA cross‐links (ICLs) and intrastrand DNA cross‐links that block DNA replication, transcription, etc., resulting in apoptosis.^6^, ^7^ Although most patients initially respond to platinum drug‐based chemotherapy and achieve remission, many patients become refractory to platinum drugs over time and ultimately succumb to the disease due to their resistance to platinum drugs.^8^ Increased DNA repair activity is one of the major mechanisms leading to platinum drug resistance in cancers.^7^ Ovarian cancer (OC) is one of the most common gynaecologic cancers with the highest mortality rate.^9^ Current treatment consists of surgery followed by platinum drug‐based chemotherapy.^10^, ^11^ However, up to 80% of OC patients eventually develop resistance to platinum drugs.^12^ Thus, it is urgent to develop novel approaches to overcome the platinum drug resistance of OC.

And‐1/WDHD1/Ctf4 is an acidic nucleoplasmic DNA‐binding protein containing N‐terminal WD40 domains, a middle SepB domain, and a C‐terminal high mobility group (HMG) domain.^13^ We and others have demonstrated that And‐1 is important for DNA replication, chromosome function, DNA damage repair, etc.^14^, ^15^, ^16^, ^17^, ^18^, ^19^ And‐1 is overexpressed in cancer cells but not in normal tissues,^20^, ^21^ suggesting And‐1 is a potential target gene for cancer therapy. To support this notion, the studies from both genetic analyses in yeast and CRISPR/Cas9 screening in human cells indicate that And‐1 is a promising cancer therapeutic target gene.^22^, ^23^ Moreover, we and others have shown that And‐1 is critical for repair of double‐stranded breaks (DSBs) by regulating HR repair,^24^, ^25^ therefore inhibition of And‐1 could be a powerful approach to increase the sensitivity of therapeutic treatments that kill cancer cells by inducing DSBs, including ionizing radiation and some chemotherapy drugs. However, so far a potent And‐1 inhibitor has yet to be found.

Resveratrol (3,5,4′‐trihydroxy‐trans‐stilbene), a phytoalexin antioxidant found in red grapes, has been reported to have the inhibitory function on multiple cancers including colon cancer, liver cancer, neuroendocrine tumor, multiple myeloma, and prostate cancer.^26^ Resveratrol targets multiple pathways, such as epidermal growth factor, transforming growth factor‐beta,^27^ protein kinase B,^28^ cyclin A2, cyclin B1,^29^ cyclin‐dependent kinases,^30^ epithelial–mesenchymal transition^31^ and ribonucleotide reductase.^32^ Moreover, resveratrol also works as an anti‐cancer regime in combination with cisplatin,^33^ gemcitabine^34^ and fluorouracil.^35^ However, resveratrol has low oral bioavailability due to its rapid metabolism, which limits its clinical applications.^36^

Bazedoxifene acetate (BZA) is an FDA approved drug for the treatment of symptoms associated with menopause and the prevention of postmenopausal osteoporosis.^37^ BZA is a selective activator of estrogen receptor and could increase bone mineral density, reduces the rate of bone turnover and decreases the risk for new vertebral fractures.^38^, ^39^, ^40^, ^41^ BZA was reported to be well tolerated, with a favorable safety profile and no evidence of endometrial or breast tissue stimulation.^42^, ^43^ A recent study found that BZA also inhibits head and neck cancer growth and metastasis via blocking IL‐6 signaling and reverses resistance to cisplatin and ionizing radiation.^44^ However, the application of BZA in the cancer treatment and inhibitory mechanism of BZA in cancers remain largely unknown.

In this study, by using an And‐1 luciferase reporter assay to screen Library of Pharmacologically Active Compounds (LOPAC) in a high throughput screening (HTS) platform, and then further screen the compound analog collection, we identified two potent And‐1 specific inhibitors, BZA and an uncharacterized compound called [(*E*)‐5‐(3,4‐dichlorostyryl)benzo[*c*][1,2]oxaborol‐1(3*H*)‐ol] (CH3). Both inhibitors directly bind and inhibit And‐1 activity by inducing And‐1 degradation. Specifically, CH3 binds to the N‐terminus of And‐1 and induces a conformational change in And‐1, which promotes the interaction of And‐1 with E3 ligase CUL4B for ubiquitin‐mediated degradation. Significantly, CH3 exhibits significant inhibition on the growth of a broad range of cancer types in NCI‐60 cell testing. Using animal tumor models, we demonstrated that And‐1 inhibitors can inhibit breast and ovarian tumor growth, and overcome platinum drug resistance of OC. Thus, our findings reveal a novel therapeutic regimen for the treatment of a broad range of cancer types.

## 2 MATERIALS AND METHODS

### 2.1 Antibodies and reagents

Antibodies used in this study are: β‐actin (5441; Sigma–Aldrich), FLAG (F7425; Sigma–Aldrich), GAPDH (G9545; Sigma–Aldrich), CUL4B (HPA011880; Sigma–Aldrich), ubiquitin (3933S; Cell Signaling Technology), cleaved caspase‐3 (9661; Cell Signaling Technology), HA‐tag (sc‐7392; Santa Cruz Biotechnology), ataxia telangiectasia and Rad3‐related protein (ATR) (sc‐1887; Santa Cruz Biotechnology), Mouse γ‐H2AX (05–636; Millipore). And‐1 antibody was previously described.^21^ Rabbit p‐And‐1 antibody was raised by using peptide C‐KAAELTA(pT)QVEEE‐amide (Thermo Fisher Scientific).

Cisplatin (479306) and BZA (PZ0018) were from Sigma–Aldrich. Cisplatin was dissolved in sterile saline for in vitro and in vivo use. ATR inhibitor VE821 was from Sigma–Aldrich (SML1415‐5MG). The synthesis method and characterization of resveratrol derivatives were shown in supporting information.

### 2.2 Cell lines and cisplatin resistant cell line establishment

The human OC cells PEO14 and PEO23 (Sigma) were cultured in RPMI‐1640 with 10% fetal bovine serum (FBS). Human ovarian carcinoma cell line (IGROV1) and IGROV1 CR cells were as described previously,^45^ and were cultured in DMEM with 10% FBS. MCF7 (ATCC) were cultured in DMEM with 10% FBS. OV90 (ATCC) were cultured in the growth medium containing 1:1 MCDB 105 (Sigma) and M199 (Sigma) supplemented with 10% FBS. All the cells were cultured at 37°C in a humidified incubator containing 5% CO_2_.

Resistant cells OV90 CR were generated using the approach as previously described.^45^ Briefly, Wild type cells OV90 were treated with cisplatin for six cycles (4 h of cisplatin treatment, followed by release to cisplatin free medium for three weeks). In the next cycle, cisplatin treatment was repeated with an increased concentration of cisplatin. After five months of treatment (6 cycles), cisplatin resistant cell OV90 CR was obtained. Only early‐passage (< 10 passages) resistant cell lines were used for the study (Figure S1A).

### 2.3 Cell viability assay

The assay was as previously described.^45^

### 2.4 And‐1 luciferase reporter assay

And‐1‐Luc reporter HEK293 cells were dispensed at 2000 cells/5 μl/well in tissue culture treated white wall/solid bottom 1536‐well plates (Greiner Bio‐One North America, Monroe, NC, USA) using a Thermo Scientific Multidrop Combi (ThermoFisher Scientific, Inc.). After the cells were incubated in assay plates at 37°C for 5 h, 23 nl of compounds (LOPAC) was transferred into the assay plates using a Wako Pintool station (Wako Automation, San Diego, CA, USA). The assay plates were incubated at 37°C for 48 h, followed by the addition of 5 μl ONE‐Glo luciferase reagent (Promega, Madison, WI, USA) using a Flying Reagent Dispenser (Aurora Discovery, Carlsbad, CA, USA). After 30 min of incubation at room temperature, the luminescence intensity of the assay plates was quantified using a ViewLux plate reader (PerkinElmer, Shelton, CT, USA). LOPAC contains 1,280 pharmacologically active and well‐validated compounds (Sigma–Aldrich).

**HIGHLIGHTS**

- 1. BZA and CH3 specifically inhibit And‐1 by promoting its degradation.
- 2. And‐1 inhibitor CH3 suppresses the growth of a broad range of cancers.
- 3. And‐1 inhibitors re‐sensitize platinum‐resistant ovarian cancer cells to platinum drugs in vitro and in vivo.

### 2.5 Clonogenic assay

Assay was conducted as previously described.^45^

### 2.6 Transfection

And‐1 siRNA was from Sigma–Aldrich. Control siGL2 and And‐1 siRNA were designed as described previously.^24^ And‐1 plasmids were designed as previously described.^24^

### 2.7 Western blotting

The assay was as previously described.^45^

### 2.8 Animal experiments

The information on animal experiments is provided in the Supporting information.

### 2.9 Ovarian cancer patients

The information on human patients is provided in the Supporting information and Table 2.

### 2.10 Cellular thermal shift assay

Cellular thermal shift assay was as previously described.^46^ Briefly, cells were incubated with MG132 (10 μM), CH3, BZA or Dimethyl sulfoxide (DMSO) for 8 h. After washing with ice‐cold PBS (suppled with Protease Inhibitor Cocktail, Roche), cells were aliquoted into PCR tubes (100 μl each) and then incubated at different temperatures (from 45 to 58°C) for 4 min. After the cells were frozen and thawed twice using liquid nitrogen, proteins were isolated from the cells after centrifugation and incubated at 58.8°C for 10 min for analysis by Western blotting.

### 2.11 NCI‐60 human tumour cell line screen

The NCI‐60 cell line panel includes 60 human tumour cell lines. The effect of CH3 on tumour cell growth was tested at National Cancer Institute, Developmental Therapeutics Program (NCI‐DTP) by following the protocol as described previously.^47^

### 2.12 *In silico* docking

Virtual protein docking was performed online at www.dockingserver.com. And‐1 protein structure was obtained from the PDB database (PDB ID: 5GVA, 5GVB, 5GOS).

### 2.13 Statistical analysis

GraphPad Prism 7.0 software was used for statistical analysis. Data were represented as the mean ± the standard error of the mean (SEM). Statistical analysis was performed using one‐way ANOVA or Student's *t* test. *p* < 0.05 was considered as significance. For Kaplan Meier survival analysis, a log‐rank (Mantel–Cox) test was used to compare each of the arms.

## 3 RESULTS

### 3.1 Identification of And‐1 inhibitors

**FIGURE 1:**
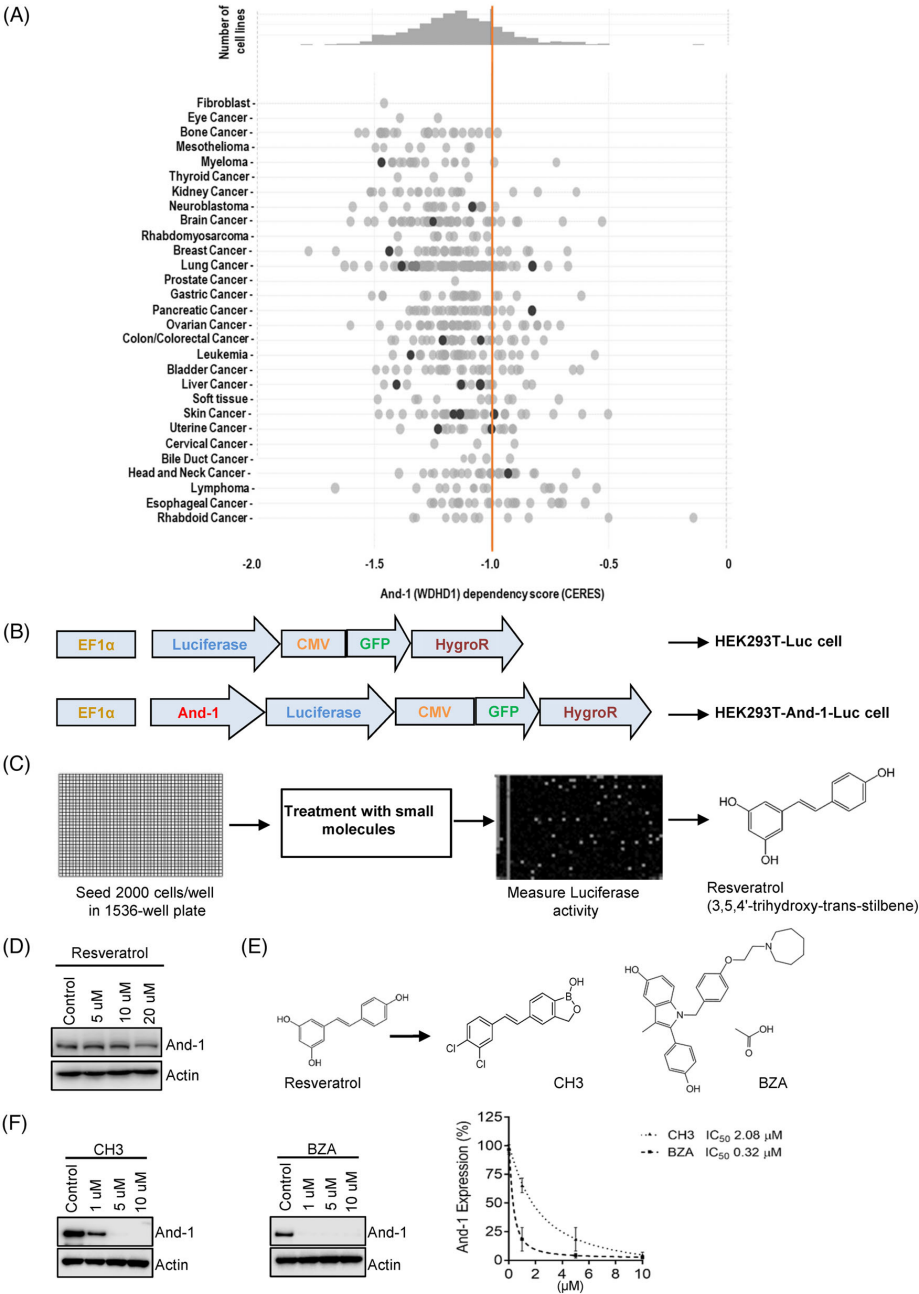
Identification of acidic nucleoplasmic DNA‐binding protein 1 (And‐1) inhibitors. (A) Dependency score of WDHD1/And‐1 analyzed by DepMap. Negative dependency score indicates the critical requirement of And‐1 by pan‐cancer cells. (B) Establishment of HEK293T‐And‐1‐Luc and HEK293T‐Luc cells for the high‐throughput screen (HTS). (C) Schematic diagram of quantitative HTS (qHTS) for And‐1 inhibitors. (D) Immunoblot analyses to detect And‐1 expression in IGROV1 cells treated with resveratrol at indicated concentrations. (E) Chemical structures of resveratrol and its three analogs as indicated. (F) Left, immunoblot analyses to detect And‐1 expression in IGROV1 cells treated with And‐1 inhibitors at the indicated concentration. Right, quantification of And‐1 expression shown in the left panel. IC_50_ was indicated

To evaluate whether And‐1 is required for tumor survival, we examined the dependence of a broad range of tumor cells on And‐1 by using dependency score analysis from DepMap (https://depmap.org/portal/). The analyses showed a highly negative dependency score of And‐1/WDHD1 in all major types of cancers, implying that And‐1/WDHD1 was essential for cell growth and proliferation across pan‐cancer cells (Figure 1A). These results further support the notion that targeting And‐1 is a promising strategy to inhibit tumors. To identify And‐1 specific pharmacologic inhibitor, we established an HTS assay to identify small molecules that can inhibit And‐1 by inducing its degradation. To this end, we first established two stable cell lines, HEK293T‐And‐1‐Luc expressing And‐1 protein fused to a luciferase reporter gene, and HEK293T‐Luc expressing luciferase only (Figure 1B). HEK293T‐And‐1‐Luc cells allow us to monitor And‐1 protein levels by measuring luciferase activity in a high‐throughput manner. Using these cell lines, we conducted a screening for And‐1 inhibitors against a LOPAC small molecule library by using a quantitative HTS (qHTS) as we described previously.^46^ From this screening, we identified resveratrol that could reduce And‐1 levels with IC_50_ at 18.91 μM (Figure 1C, Table S1). Using IGROV1 cells, we confirmed that resveratrol inhibited And‐1 expression with a relatively high IC_50_ value (16.70 μM) (Figure 1D). The high IC_50_ of resveratrol raised the off‐target concerns, which may compromise the potential clinical application of And‐1 inhibitors. We, therefore, screened for more potent And‐1 inhibitors from a compound collection of resveratrol derivatives or analogs (Figure S1B). From this screen, we found two compounds, CH3 and BZA, which reduced And‐1 expression level in IGROV1 cells with IC_50_ of 2.08 μM, and 0.32 μM, respectively (Figure 1E,F).

And‐1 is involved in DNA replication in the S‐phase and is required for the S‐phase progression.^15^ We assumed that And‐1 inhibitors should be able to inhibit DNA replication by targeting And‐1. Indeed, FACS analyses indicated that both CH3 and BZA reduced the percentage of cells in the S‐phase in a manner similar to those in And‐1 depleted cells (Figure S1C). Previous studies showed that that And‐1 is required for homologous recombination (HR) repair by promoting the recruitment of CtIP to DSB sites.^24^, ^25^ Consistently, CH3 and BZA treatment significantly decreased the recruitment of CtIP to DSB sites induced by micro‐irradiation (Figure S1D). Furthermore, the inhibition of And‐1 by CH3 or BZA profoundly reduced HR efficiency (Figure S1E,F). Together, these data clearly demonstrated that CH3 and BZA significantly inhibit expression levels of And‐1.

### 3.2 And‐1 inhibitors interact with And‐1

**FIGURE 2:**
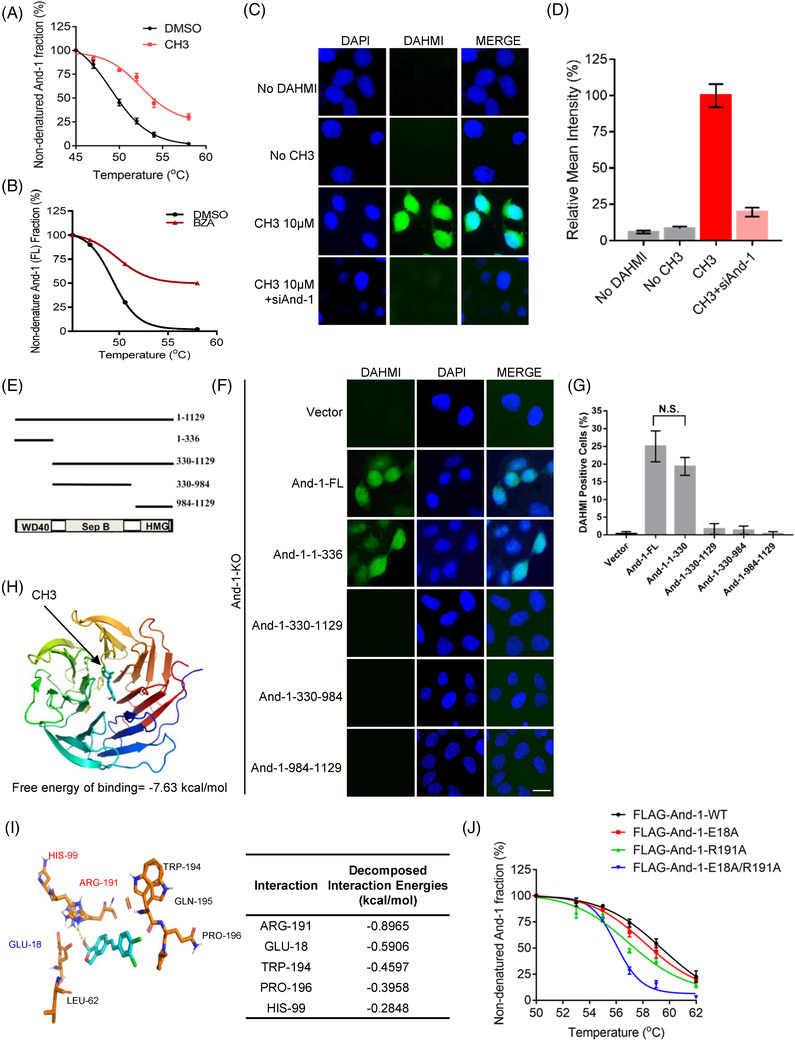
Acidic nucleoplasmic DNA‐binding protein 1 (And‐1) inhibitors directly interact with And‐1. (A and B) Thermal stability of And‐1 in IGROV1 cells treated with CH3 (A) and bazedoxifene acetate (BZA) (B). (C) Left, DAHMI staining assay to detect the distribution of CH3 in live IGROV1 cells. siRNA was given to cells 24 h before CH3 treatment. DAHMI was added to the cells 24 h after CH3 treatment. (D) Quantification of fluorescent intensity in cells analyzed in C. The intensity was normalized to cells treated with 10 μM of CH3. (E) A schematic of domains of And‐1. (F) DAHMI staining assay to detect CH3 distribution in live And‐1 knockout cells expressing ectopic And‐1 or its truncation mutants. (G) Quantification of fluorescent intensity of cells shown in F. (H) Docking pose of CH3 (green) in WD40 domain of And‐1 (rainbow). (I) Left, amino acids of And‐1 contributing to the interaction with CH3. Right, the decomposed interaction energy of top 5 amino acids of And‐1 contributing to interaction with CH3. (J) Thermal stability of FLAG‐And‐1‐WT, FLAG‐And‐1‐E18A, FLAG‐And‐1‐R191A and FLAG‐And‐1‐E18A/R191A in IGROV1 cells treated with CH3

To explore whether And‐1 inhibitors directly bind to And‐1, we conducted the cellular thermal shift assay, which could detect the direct interaction between a compound and protein because a small molecule results in thermal stabilization of the bound protein.^48^ From this analysis, we found that at higher temperature And‐1 protein was stabilized upon treatment with CH3 or BZA (Figure 2A,B, Figure S2A,B) compared to the DMSO treatment. The boron‐containing compounds could be visualized by a specific fluorescent boron sensor Boronic acid sensor (DAHMI) in live cells.^49^ Given that CH3 contains a boron group and And‐1 is a nuclear protein, we assumed that if And‐1 is a primary target of CH3, CH3 could be visualized by DAHMI in the nuclei of cells treated with CH3 and And‐1 depletion should reduce the amount of CH3 in the nuclei. Indeed, CH3 was detected as indicated by the fluorescent signal in nuclei 1 min after the DAHMI treatment in IGROV1 cells, and And‐1 depletion by siRNA significantly reduced the intensity of the fluorescent signal in nuclei (Figure 2C and D), indicating that And‐1 is a primary target of CH3. Consistently, overexpression of And‐1 in three different cells can rescue the viability of cells treated by CH3 and BZA (Figure S2C).

To identify the CH3‐binding region on And‐1, we used the DAHMI system to analyze the binding affinity of CH3 in And‐1 knockout U2OS cells ectopically expressing wild‐type And‐1 or its mutants, including And‐1(1‐330) containing WD40 domain, And‐1 (330–1129) containing SepB and HMG domains, And‐1 (330–984) containing Sep domain, and And‐1 (984–1129) containing HMG domain (Figure 2E). As shown in Figure 2F and G, wild‐type And‐1 and And‐1(1‐330) but no other And‐1 mutants exhibited binding affinity to CH3, suggesting CH3 binds to the N‐terminal region of And‐1. Consistently, by analyzing binding energy between CH3 and And‐1 domains using virtual ligand‐protein docking,^50^ we found that the WD40 domain is the potential binding domain for CH3 because it exhibited the lowest free energy of binding (Figure 2H). We further examined the binding energy of each binding site on And‐1 with CH3 and found that Arginine‐191 and Glutamic acid‐18 had the lowest interaction energy with CH3 (Figure 2I), suggesting both amino acids are potential critical binding sites for these inhibitors. Indeed, the thermo shift assay indicated that either R191A or E18A mutations have minor effects on And‐1 thermostability, but mutation of both R191a and E18A significantly impaired the thermostability of And‐1 in response to the CH3 treatment (Figure 2J, Figure S2D–G). Together, these results indicate that CH3 directly binds to the WD40 domain of And‐1. Molecular docking analysis indicated that BZA also bound to And‐1 WD40 domain and And‐1 mutants without WD40 domain were no longer degraded in response to BZA (Figure S2H,I).

### 3.3 CH3 induces And‐1 degradation via CUL4B‐mediated proteasome degradation pathway

**FIGURE 3:**
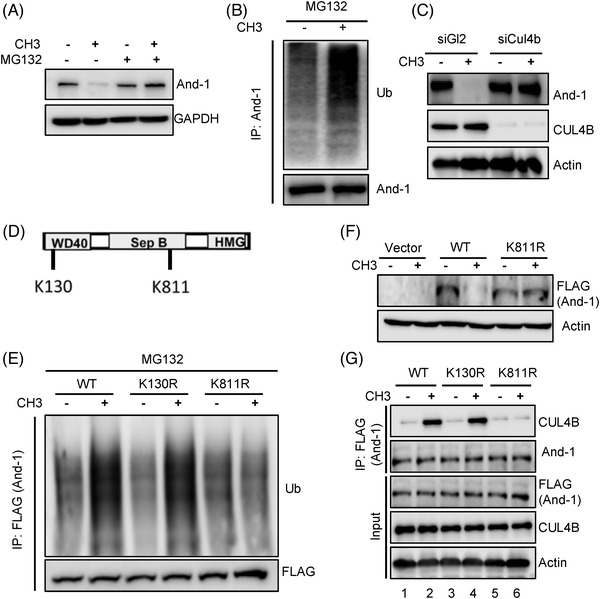
CH3 induces acidic nucleoplasmic DNA‐binding protein 1 (And‐1) degradation via the CUL4B‐mediated proteasome degradation pathway. (A) Immunoblot analyses to detect the And‐1 expression in IGROV1 cells treated with 5 μM CH3 for 48 h followed by 10 μM of MG132 treatment for 4 h before harvesting. (B) Immunoblot analyses to detect ubiquitinated And‐1 in IGROV1 cells treated with CH3. Cell lysates were subjected to immunoprecipitation with antibodies against And‐1. (C) Immunoblot analyses to detect the And‐1 expression in CUL4B knockdown IGROV1 cells treated with CH3. siRNAs were transfected 24 h before exposure to CH3 for an additional 48 h. (D) Schematic of predicted ubiquitination sites on And‐1. (E) Immunoblot analyses to detect the ubiquitinated FLAG‐And‐1‐WT, FLAG‐And‐1‐K130R or FLAG‐And‐1‐K811R in IGROV1 cells treated with CH3. IGROV1 cells transfected with indicated plasmids were treated with CH3, followed by 10 μM of MG132 treatment. Cell lysates were prepared and subjected to immunoprecipitation with antibodies against FLAG. (F) Immunoblot analyses to detect expression of FLAG‐And‐1‐WT or FLAG‐And‐1‐K811R in IGROV1 cells treated with CH3. (G) Immunoblot analyses to detect the interactions of CUL4B with FLAG‐And‐1‐WT, FLAG‐And‐1‐K130R or FLAG‐And‐1‐K811R in cells treated with or without CH3, followed by 10 μM of MG132 treatment

We next sought to investigate the molecular mechanism by which And‐1 inhibitors induce And‐1 degradation. To this end, we first determined whether the ubiquitin‐mediated proteasome pathway was involved in CH3‐induced And‐1 degradation. To this end, IGROV1 cells were treated with CH3 in the absence or the presence of 26S proteasome inhibitor MG132. As shown in Figure 3A, the CH3 treatment reduced And‐1 protein levels, and reduced And‐1 levels were restored by MG132 treatment, suggesting that CH3‐induced And‐1 degradation is regulated by the proteasome pathway. Protein degradation through the proteasome requires conjugation of one or more ubiquitin molecules to the target.^51^ To address whether And‐1 is modified by ubiquitin in IGROV1 cells treated with CH3, And‐1 protein was immunoprecipitated and probed with the anti‐ubiquitin antibody. As shown in Figure 3B, the discrete slower‐migrating ubiquitinated And‐1 bands were increased in cells treated with CH3, indicating that CH3 induces And‐1 degradation through the ubiquitin‐mediated proteasome pathway.

The above observations encouraged us to identify E3 ubiquitin ligase targeting And‐1 for the degradation by CH3. A previous study in yeast cells indicated that E3 ligase Rtt101 interacts with Ctf4/And‐1.^52^ Since the human analog of Rtt101 is CUL4,^53^ we predicted that CUL4 might be the E3 ligase responsible for And‐1 degradation. Indeed, knockdown of CUL4B by siRNA dramatically restored And‐1 protein levels in cells treated with CH3, indicating And‐1 is a substrate of CUL4B for ubiquitination by CH3 (Figure 3C). To determine the ubiquitination sites of And‐1, we used the proteomic analysis program (https://www.nextprot.org/entry/NX_O75717/proteomics) and identified Lysine 130 and Lysine 811 as potential ubiquitination sites. Strikingly, mutation K811R but not K130R abolished And‐1 ubiquitination induced by CH3 (Figure 3D,E), indicating that K811 was a *bona‐fide* ubiquitin site. Consistently, FLAG‐And‐1‐K811R mutant was not degraded in cells treated with CH3 (Figure 3F). In agreement with the notion that CUL4B mediated the degradation of And‐1, CH3 treatment increased the interaction between FLAG‐And‐1 and CUL4B (Figure 3G, lanes 1 and 2). Interestingly, the interaction between CUL4B and FLAG‐And‐1‐K811R was not induced by CH3 compared to FLAG‐And‐1‐WT and FLAG‐And‐1‐K130R, indicating Lysine 811 of And‐1 is required for CH3‐induced interaction between And‐1 and CUL4B (Figure 3G, lanes 5 and 6).

### 3.4 CH3 promotes the interaction between And‐1 and CUL4B by altering And‐1 conformation

**FIGURE 4:**
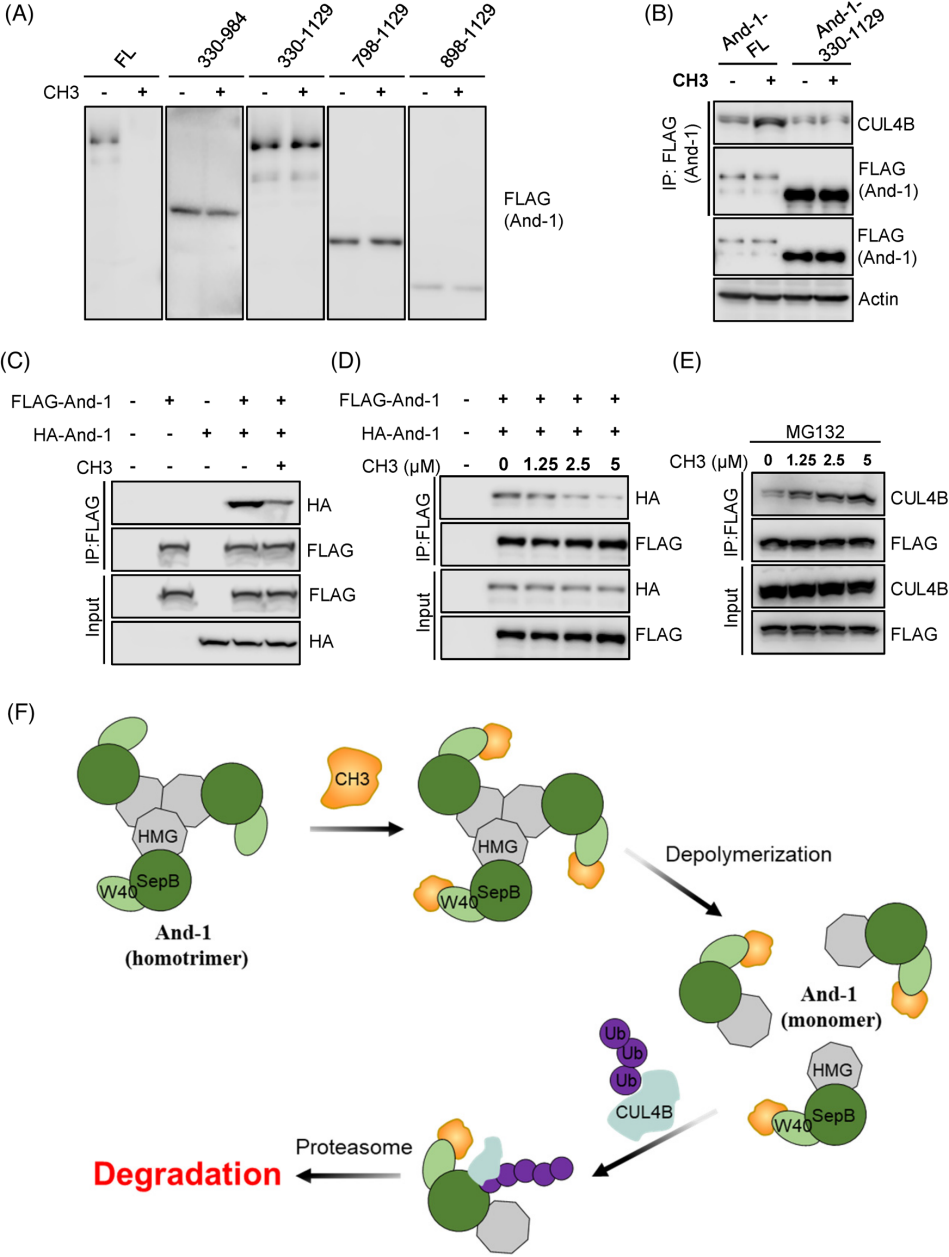
CH3 promotes the interaction of acidic nucleoplasmic DNA‐binding protein 1 (And‐1) with CUL4B by altering the And‐1 conformation. (A) Immunoblot analyses to detect the expression of full‐length FLAG‐And‐1 and its truncation mutants in cells treated with or without CH3. (B) Immunoblot analyses to detect the interactions of CUL4B with FLAG‐And‐1‐WT or FLAG‐And‐1‐330‐1129. (C) Immunoprecipitation analyses to detect the interaction of HA‐And‐1 with FLAG‐And‐1 in cells treated with or without CH3. (D) Immunoprecipitation analyses to detect the interaction of HA‐And‐1 with FLAG‐And‐1 in cells treated with CH3 at indicated concentrations. (E) Immunoprecipitation analyses to detect the interaction of And‐1 with CUL4B in cells treated with CH3 at indicated concentrations in the presence of MG132. (F) Model to show how CH3 promotes the interaction of And‐1 with CUL4B by altering And‐1 conformation. See the text for detail

Given that the WD40 domain mediates the interaction of And‐1 with CH3, we assumed that And‐1 mutants without WD40 should not be degraded by CH3. Indeed, unlike wild‐type And‐1, mutants And‐1‐330‐984, And‐1‐330‐1129, And‐1‐798‐1129 and And‐1‐898‐1129 were not degraded in response to CH3 (Figure 4A). Thus, the WD40 domain of And‐1 is required for And‐1 degradation by CH3. We next tested whether the WD40 domain is required for increased And‐1‐CUL4B interaction induced by CH3. As shown in Figure 4B, CH3 induced the interaction of CUL4B with full‐length And‐1 but not And‐1‐330‐1129, indicating the CH3‐binding domain WD40 is required for CH3‐induced interaction of And‐1 with CUL4B.

Human And‐1 exists as a homotrimer mediated by the SepB domain,^54^ we therefore hypothesized that the interaction of CH3 with WD40 domain may disrupt And‐1 polymerization, resulting in exposure of CUL4B binding site on And‐1 to CUL4B. To test this hypothesis, we transfected both FLAG‐And‐1 and HA‐And‐1 in U2OS cells and examined the formation of homotrimer by measuring the interaction of FLAG‐And‐1 with HA‐And‐1 in the presence of CH3. As shown in Figure 4C,D, the CH3 treatment significantly decreased the interaction between HA‐And‐1 and FLAG‐And‐1 in a CH3‐dose‐dependent manner, suggesting that CH3 indeed disrupts the polymerization of And‐1. We therefore hypothesized that de‐polymerization of And‐1 by CH3 may promote the interaction of And‐1 with CUL4B. Indeed, CH3 induced the interaction of FLAG‐And‐1 with CUL4B in a CH3‐dose‐dependent manner in the presence of MG132 (Figure 4E). Thus, CH3‐induced depolymerization of And‐1 promotes the interaction of And‐1 with CUL4B, resulting in degradation of And‐1 via ubiquitin‐mediated proteasome pathway (Figure 4F).

### 3.5 CH3 exhibits the significant inhibition in a broad range of cancer cells in vitro and in vivo

**FIGURE 5:**
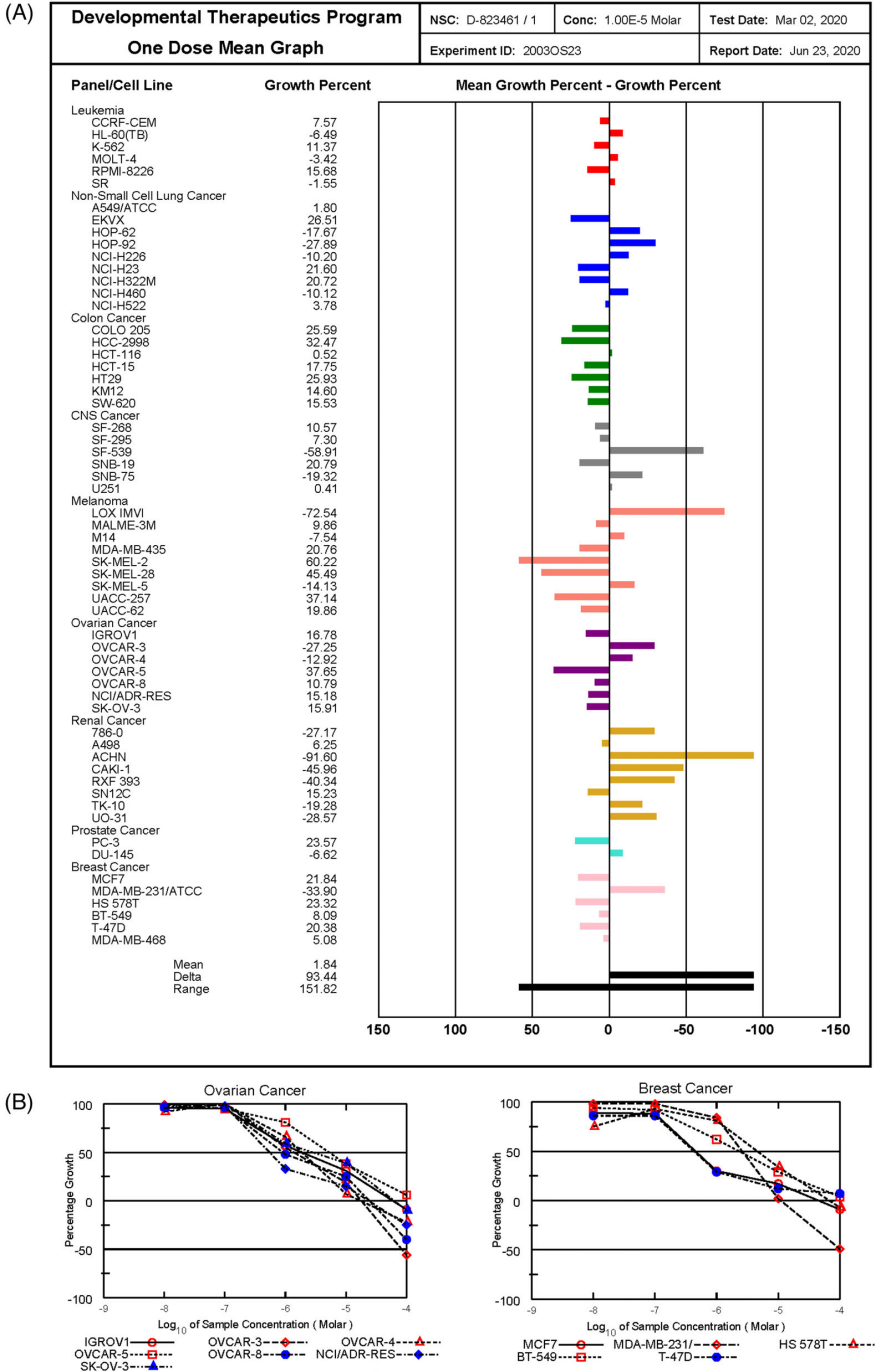
The effect of CH3 treatment in the NCI‐60 human tumor cell line panel. (A) Mean graph display of NCI‐60 cell line screening data for CH3. The presented result is a one‐dose mean graph display of NCI‐60 cell line screening data for CH3 at 10 μM. The number reported for the one‐dose assay is growth relative to the no‐drug control. Bars deflecting to the right of the mean indicate relatively high sensitivity, and bars deflecting to the left of the mean indicate relatively low sensitivity to CH3. Experiments were performed at the NCI‐DTP. (B) Dose–response curves of CH3 for breast and ovarian cell lines from NCI‐60 cell line panel. All cells were tested at the NCI‐DTP in the presence of CH3 at five concentrations (0.01 μM, 0.1 μM, 1 μM, 10 μM and 100 μM). Dose–response curves for all other tested cell lines were shown in Figure S3

We next evaluate the inhibitory efficacy of CH3 against a panel of 60 human cancer cell lines at the NCI‐DTP. The one dose anti‐cancer assay, which reported the inhibitory activity of compound as a mean graph of the percent growth of treated cells compared to the untreated control cells, showed that CH3 significantly reduced cancer cell growth with a mean growth percentage to 1.84% at 10 μM against all 60 tumor cell lines (Figure 5A). To further compare the efficacy of CH3 in the inhibition of NCI 60 cells, we examined the inhibitory effects of CH3 on 60 cells by using five doses of CH3. CH3 was a highly active inhibitor of cell growth and demonstrated with low growth inhibition of 50 (GI_50_) and total growth inhibition (TGI) values (Figure 5B, Figures S3A,B). CH3 was more effective against renal, leukemia and central nervous system cancers (Figure S3B). Taken together, the results suggest that CH3 is a promising anticancer agent with great potential against a broad range of cancer types.

**FIGURE 6:**
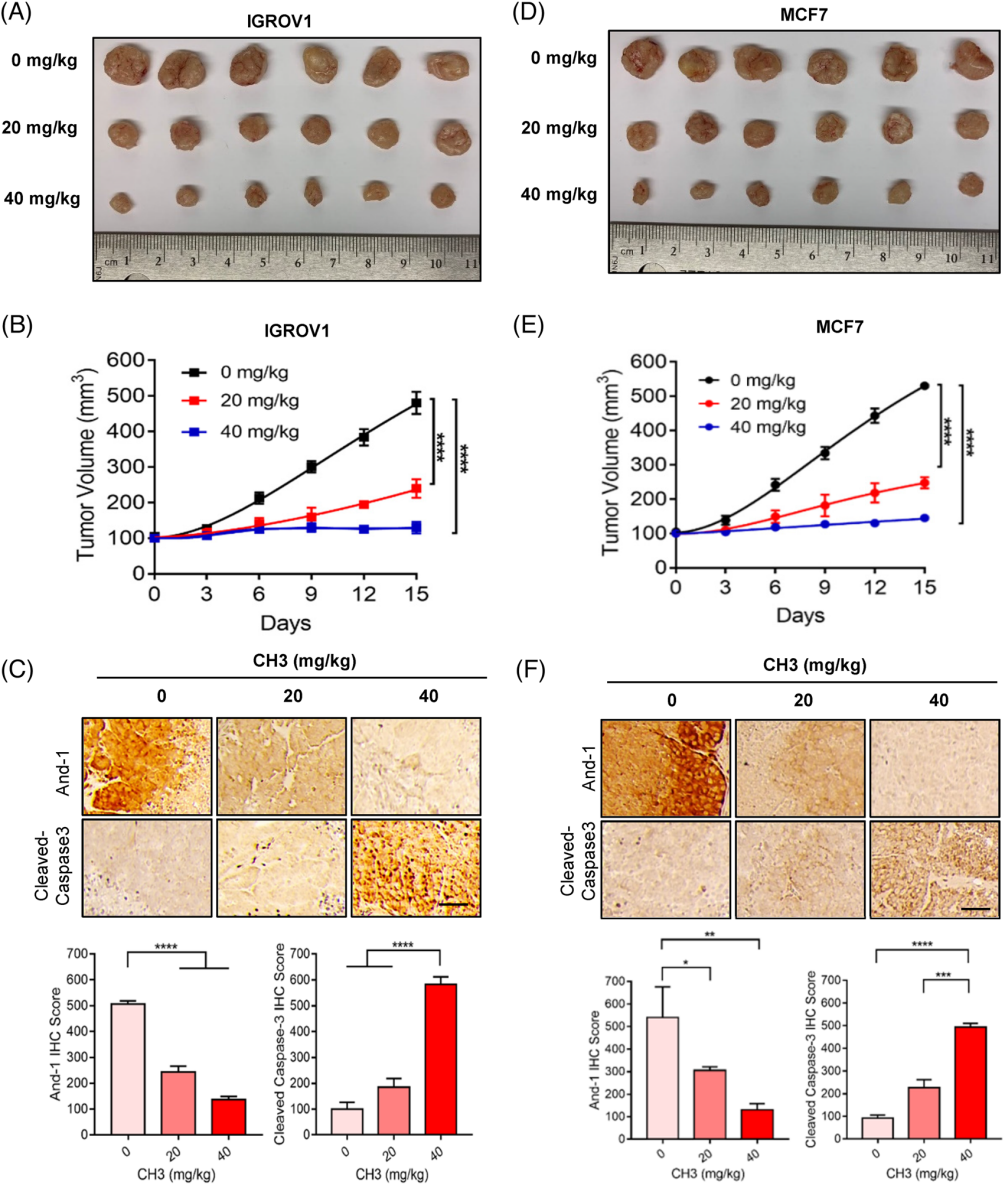
CH3 significantly inhibits tumor growth in vivo. (A) Tumours of IGROV1 xenograft treated with vehicle or CH3 (20 or 40 mg/kg/3days intraperitoneally) for 3 weeks. Ruler scale is in centimeters. (B) Growth curves of IGROV1 xenografts. Data are represented as means ± SEM. ****p < 0.0001. (C) IHC staining against acidic nucleoplasmic DNA‐binding protein 1 (And‐1) and cleaved‐caspase3 antibodies of tumor samples from 3 groups of mice. Scale bar: 50 μm. Lower panel, quantification of And‐1and cleaved‐caspase 3 IHC score in tumors treated in A. ****p < 0.0001. (D) Tumors of MCF7 xenograft treated with vehicle or CH3 (20 or 40 mg/kg/3days intraperitoneally) for 3 weeks. Ruler scale is in centimeters. (E) Growth curves of MCF7 xenografts. Data are represented as means ± SEM. ****p < 0.0001. (F) IHC staining against And‐1 and cleaved‐caspase3 antibodies of tumor samples from three treated groups of mice. Scale bar: 50 μm. Lower panel, quantification of And‐1and cleaved‐caspase 3 IHC score in tumors treated in (D). *p < 0.1; **p < 0.01; ***p < 0.001; ****p < 0.0001

To evaluate the potency of And‐1 inhibitors on tumors in vivo, we selected two types of cancer cell lines OC IGROV1 and breast cancer MCF7 cells, which were also used for NCI‐60 cell line screen. To this end, we subcutaneously implanted IGROV1 or MCF7 cells into nude mice to form ovarian or breast tumors, respectively. The mice with these tumors were then treated with vehicle, 20 mg/kg or 40 mg/kg of CH3 via intraperitoneal injection. As shown in Figure 6A,B,D, E, CH3 significantly reduced tumor growth of ovarian IGROV1 and breast MCF7 xenografts at both treated doses. The immunohistochemistry (IHC) analyses of tumor tissues for expression levels of And‐1 and cleaved caspase‐3 antibodies demonstrated that CH3‐inhibited And‐1 expression and induced the expression of cleaved caspase‐3, which indicated the apoptosis in treated ovarian and breast tumors (Figure 6C, F). We next evaluated the toxicity of CH3 by measuring the body weight of all treated mice and examined multiple organs, including heart, liver, kidney and lung through H&E staining, and did not observe systemic toxicity or obvious side effects in these evaluated organs (Figure S4A–D). These results suggest that CH3 is a potent and safe compound for the treatment of various types of cancer.

### 3.6 And‐1 inhibitors overcome cisplatin resistance in ovarian cancer

**FIGURE 7:**
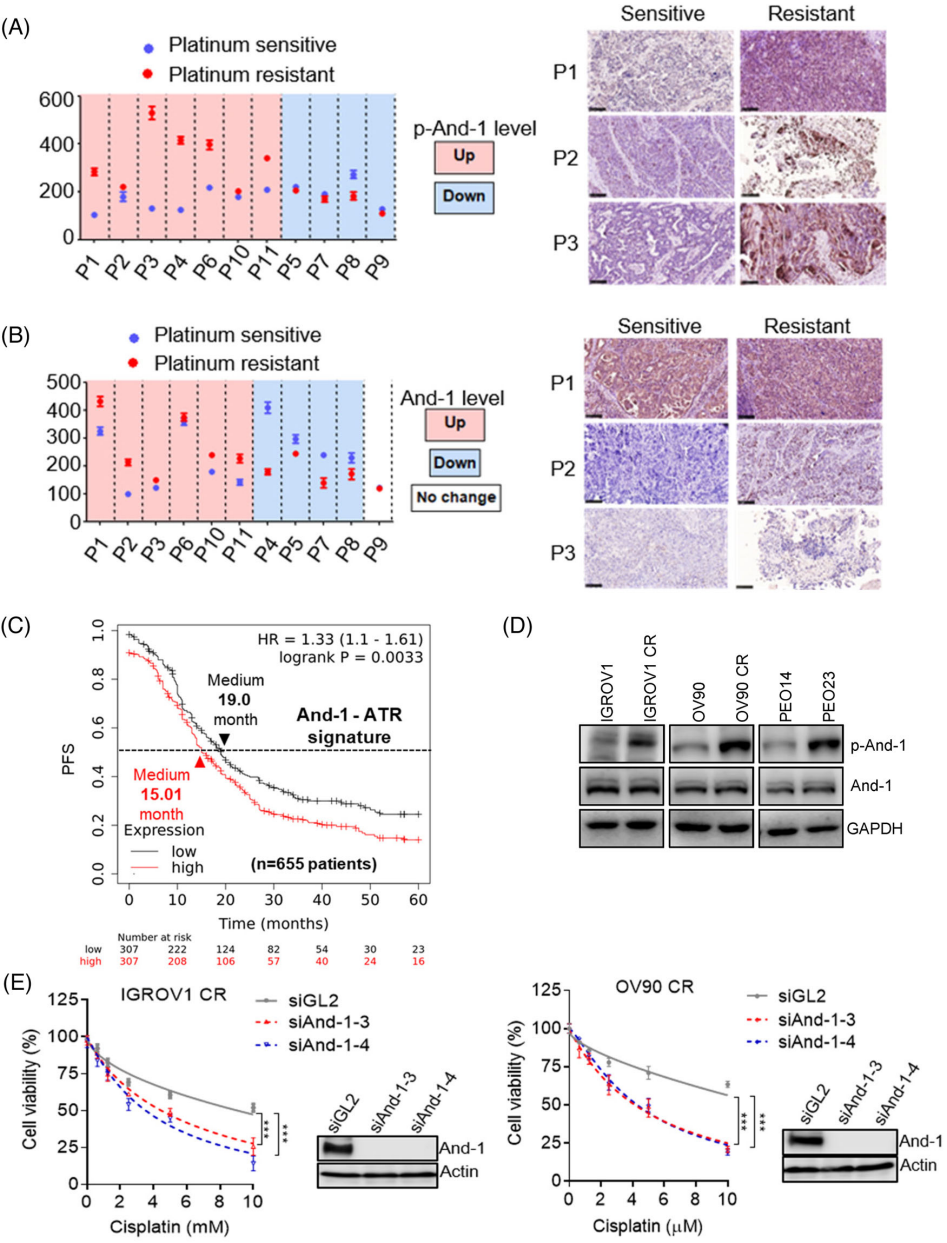
Acidic nucleoplasmic DNA‐binding protein 1 (And‐1) activation contributes to cisplatin resistance in OC. (A) IHC to examine expression levels of p‐And‐1 in tumors from ovarian cancer patients. Left, quantification of p‐And‐1 levels by IHC in tumors from the same patients (11 patients) before and after the development of platinum resistance. Data are represented as mean ± SEM. Right, representative IHC images of p‐And‐1 from the platinum drug sensitive and resistant tumor samples (P1‐P3). (B) IHC to examine expression levels of And‐1 in tumors from ovarian cancer patients. Left, quantification of And‐1 levels by IHC in tumors from the same patients (11 patients) before and after the development of platinum resistance. Data are represented as mean ± SEM. Right, representative IHC images of p‐And‐1 from the platinum drug sensitive and resistant tumor samples (P1‐P3). (C) Kaplan–Meier analyses of 5‐year progression free survival (PFS) based on clinical and molecular data for OC patients (n = 655). The patients were stratified by the expression levels in their tumors of the And‐1‐ATR signature genes. (D) Immunoblot analyses of the p‐And‐1 expression in 3 paired parental and cisplatin resistant OC cells. (E) Sulforhodamine B (SRB) assay to detect cell viability to cisplatin in IGROV1 CR or OV90 CR cells treated with indicated siRNAs

Cisplatin causes cell death by introducing DNA cross‐links that inhibit DNA replication and transcription.^7^ Increased ICL repair activity is a major mechanism to cause cisplatin resistance.^7^ In a separated study, we found that And‐1 is activated via phosphorylation at its T826 site by ATR and activated And‐1 is critical for ICL repair and cisplatin resistance in platinum drug resistant OC (manuscript submitted). To explore the potential clinical application of And‐1 inhibitors to treat platinum drug resistant OC, we used a phospho‐specific antibody that recognizes phosphorylation of And‐1 at T826 to examine expression levels of phospho‐And‐1 at T826 (p‐And‐1) in tumors from the same patient before and after the development of acquired platinum‐resistance (Table S2). We collected tumors samples from total 11 patients and examined expression levels of And‐1 and p‐And‐1 by IHC using the approach as we have described previously.^45^ Strikingly, p‐And‐1 level was increased in 7 out of 11 patients and And‐1 level was upregulated in 6 out of 11 patients after developing platinum drug resistance (Figure 7A,B), suggesting that And‐1 is activated in most of tested platinum drug resistant OC patients.

To evaluate the role of And‐1 phosphorylation by ATR in platinum drug resistance of OC patients, we used PathwayNet analysis (http://pathwaynet.princeton.edu/) to identify the top 100 genes involved in the And‐1‐mediated pathway and the top 100 genes involved in ATR‐mediated pathways. Among them, 23 genes are involved in both And‐1‐ and ATR‐mediated pathways, which were named as And‐1‐ATR signature genes (Table S3). Strikingly, OC patients with platinum drug treatment history exhibited a worse five‐year progression free survival (PFS) when And‐1‐ATR signature gene expression in the tumors was higher (Figure 7C), suggesting the ATR‐And‐1 pathway may play a critical role in platinum‐resistance of OC. To test whether the And‐1 inhibitor could overcome platinum‐resistance of OC, we examined the expression levels of p‐And‐1 and effects of And‐1 inhibition in three paired cisplatin sensitive and resistant OC cells. Significantly, p‐And‐1 levels were increased in all three resistant cells (Figure 7D), and inhibition of And‐1 by siRNA re‐sensitized the resistant cells IGROV1 CR and OV90 CR and parental cells to cisplatin (Figure 7E, Figure S4E), which are consistent with our separated studies in different paired cell lines (manuscript submitted). Together, these data strongly suggest that the inhibition of And‐1 is a potential novel approach to treat platinum drug resistance in OC.

**FIGURE 8:**
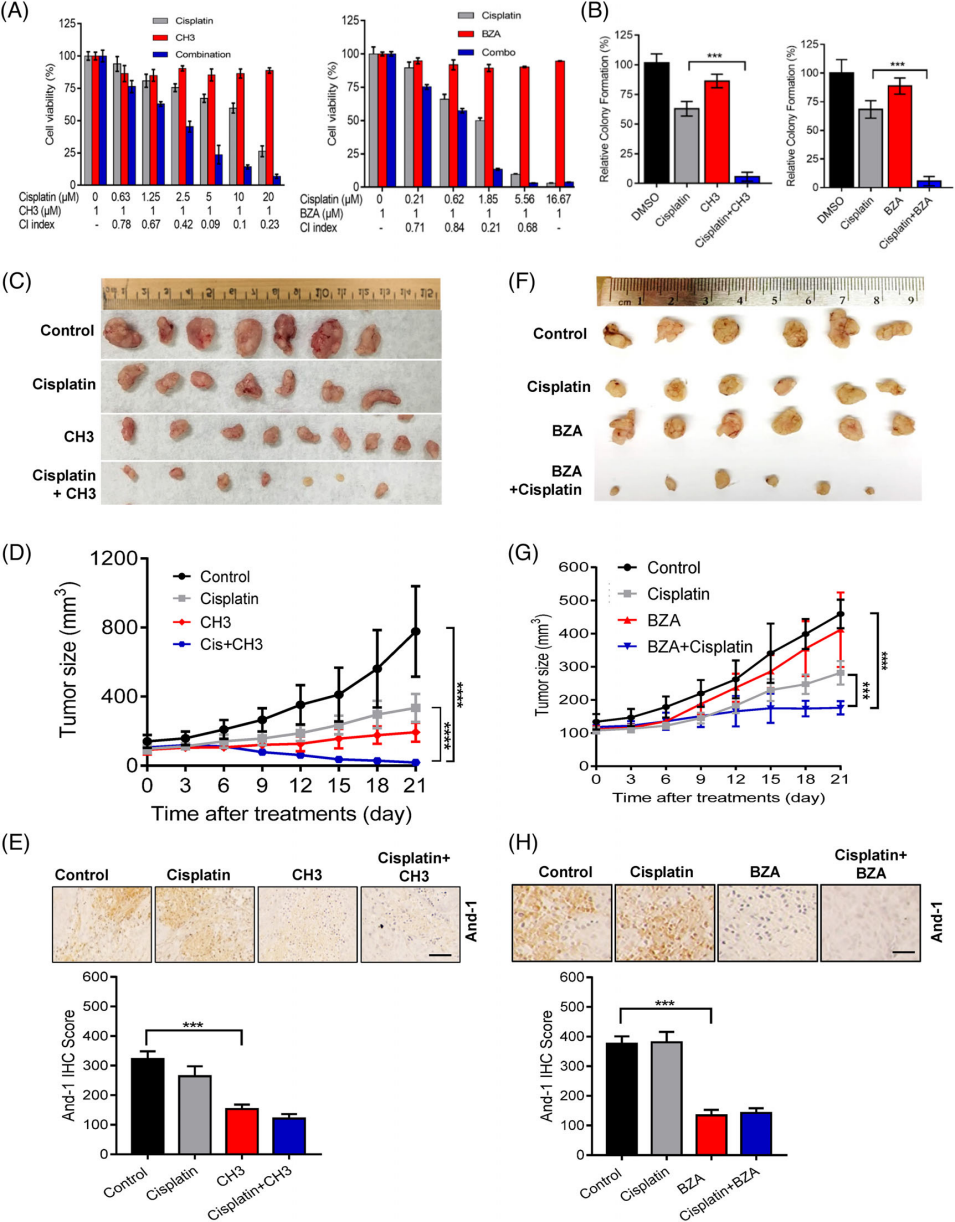
Acidic nucleoplasmic DNA‐binding protein 1 (And‐1) inhibitors overcome cisplatin resistance in ovarian cancer (OC). (A) SRB assay to detect cell viability to cisplatin in IGROV1 CR cells treated with CH3 (left) or bazedoxifene acetate (BZA) (right). Synergy of each combination was indicated as combination index (CI) values. (B) Colony formation in IGROV1 CR cells treated with DMSO, cisplatin, CH3 or combination of cisplatin and CH3 (left), or treated with DMSO, cisplatin, BZA or combination of cisplatin and BZA (right). (C) Tumors of IGROV1 CR xenograft treated with control (vehicle), cisplatin (8 mg/kg/day intraperitoneally), CH3 (20 mg/kg/3days intraperitoneally), or a combination of CH3 and cisplatin for 3 weeks. Ruler scale is in centimeters. (D) Growth curves of IGROV1 CR xenografts. Data are represented as means ± SEM, ****p < 0.0001. (E) IHC staining against And‐1 antibodies of tumor samples from four treated groups of mice. Scale bar: 50 μm. Lower, quantification of And‐1 IHC score in tumor samples from mice treated with indicated drugs. ***p < 0.001. (F) Tumors of IGROV1 CR xenograft treated with control (vehicle), cisplatin (8 mg/kg/day intraperitoneally), BZA (2 mg/kg/3days intraperitoneally), or a combination of BZA and cisplatin for 3 weeks. Ruler scale is in centimeters. (G) Growth curves of IGROV1 CR xenografts. Data are represented as means ± SEM. ***p < 0.001; ****p < 0.0001. (H) IHC staining against And‐1 antibodies of tumor samples from four treated groups of mice. Scale bar: 50 μm. Lower, quantification of And‐1 IHC score in tumor samples from mice treated with indicated drugs. ***p < 0.001

To determine whether And‐1 inhibitors can overcome cisplatin resistance in OC cells, we first examined the synergy of CH3 or BZA with cisplatin in IGROV1 CR cells. As shown in Figure 8A, both CH3 and BZA exhibited significant synergy with cisplatin to inhibit cell proliferation in IGROV1 CR cells as indicated by the combination index (CI) (synergism: CI < 1; additive effect: CI = 1; and antagonism CI > 1). Consistently, the clonogenic assays demonstrated that CH3 and BZA displayed great synergy with cisplatin to reduce the clonogenic capacity of IGROV1 CR cells (Figure 8B, Figure S5A,B).

To assess the efficacy of the combination of And‐1 inhibitors and cisplatin in vivo, we treated cisplatin resistant IGROV1 CR xenografts with vehicle, And‐1 inhibitors, cisplatin or a combination of cisplatin and And‐1 inhibitor via intraperitoneal injection. Compared to the cell‐based assay result, the combinational treatments of cisplatin with CH3, or BZA significantly reduced resistant tumour growth as compared to cisplatin alone treatment (Figure 8C,D,F,G). IHC analysis showed that both CH3 and BZA suppressed expression of And‐1 in tumours, indicating that CH3 and BZA indeed inhibit And‐1 in vivo (Figure 8E,H). Importantly, these treatments had no significant effects on body weight and organs including heart, liver, kidney, and lung, indicating that the combination of cisplatin with CH3 or BZA had no obvious systemic toxicity or side effect (Figure S5E,F). Together with our results suggest that inhibition of And‐1 by its inhibitors is a promising therapeutic approach to overcome platinum drug resistance in OC.

## 4 DISCUSSION

In this study, we have identified two potent And‐1 inhibitors that exhibited great suppression on a broad range of cancer cells in vitro and in vivo. Furthermore, we elucidated the molecular mechanism of how And‐1 inhibitors induce the degradation of And‐1. Moreover, we presented the evidence to demonstrate that the inhibition of And‐1 by its inhibitors is a promising approach to overcome platinum drug resistance in OC in vitro and in vivo. These discoveries provide us with a potential novel approach to treat multiple cancers, as well as platinum drug resistant OC.

We are not surprised that And‐1 inhibitors show great inhibitory effects on a broad range of cancer types given that And‐1 is essential for cell growth and proliferation across pan‐cancer cells (Figure 1A). It is consistent with the fact that many first‐line chemotherapeutic drugs kill various types of cancers by inducing DNA damage and inhibiting DNA replication, such as cisplatin, doxorubicin, camptothecin derivatives and temozolomide.^55^ Radiotherapy and some chemotherapies kill cancer cells by inducing DNA damage, particularly DNA DSBs.^56^, ^57^ Interestingly, we and others have reported that And‐1 is critical for HR repair of DSBs.^13^, ^25^ Therefore, we expect that And‐1 inhibitors could also serve as a sensitizer to significantly increase the sensitivity of radiotherapy and chemotherapeutic drugs, and overcome the resistance to these treatments due to the increased HR repair. Further in vitro and in vivo studies are expected to test this potential application in the future.

To date, the only report on And‐1/Ctf4 inhibitor is from a rational design to disrupt the interaction of Ctf4 with DNA polymerase α by peptides.^58^ However, the best peptide candidates identified from this study exhibit IC_50_ at the micromolar range and its ability to inhibit And‐1 was not documented. In comparison, the structures of our inhibitors are completely different from inhibitors of this study (Figure 1E), and our screening and chemical modifications have resulted in the discovery of drug‐like molecules with high potency and specificity towards And‐1. CH3 and BZA displayed little toxicity in the in vivo study. CH3 is a new compound and further in vivo studies should be performed to evaluate its short and long‐term toxic effects. BZA is a FDA approved drug and in all preclinical in vivo studies, BZA was well tolerated and was not found to cause any adverse effects on plasma lipids or reproductive tract histology.^59^ Inhibiting by CH3 leads to growth inhibition of the wide range of cancers, suggesting that CH3 may provide with a potential application for the treatment of multiple types of cancer.

BZA is a novel selective estrogen receptor modulator (SERM) with activity distinct from other members of the SERM family, such as tamoxifen and raloxifene. BZA was shown to interact with the ERs, transactivate the ER, and positively affect the skeletal and lipid profile without stimulating the uterine endometrium, causing breast cancer cell proliferation or negatively impacting the central nervous system in preclinical models.^60^ Resveratrol and CH3 share similar stilbene‐like cores that are a common structural motif responsible for ERα binding. The 2‐phenyl‐3‐methyl indole core of BZA functions in a similar manner as other selective estrogen stilbene‐like cores.^61^ The side chains of these compounds are the fundamental determinant of target specificity.^62^ BZA and CH3 interact with And‐1 at WD40 domain, which possesses a different binding pocket compared to ER, suggesting that CH3 and BZA may bind to And‐1 via its side chains. Indeed, our virtual docking profiles of compound‐And‐1 interaction suggest that polar groups but not stilbene‐like cores of BZA and CH3 contribute to the majority of the binding energy at WD40 domain. Thus, the way of BZA to bind And‐1 appears to be different from its interaction with ER.

In this study, we identified two potent And‐1 inhibitors that directly bind to And‐1 protein and induce its degradation. We not only validated the specificity of these inhibitors but also revealed the inhibitory function of these compounds on a broad range of cancers. These results provide evidence for the development of a novel class of molecules by inhibiting And‐1 for the treatment of multiple cancers, as well as cisplatin resistant OC. Given that BZA is an FDA‐approved drug, we expect a clinical trial to treat cancers by re‐purposing BZA in the near future.

## 5 CONCLUSIONS

In summary, using an HTS platform, we identified two novel potent And‐1 inhibitors, BZA and CH3, which specifically inhibit And‐1 by promoting its degradation. And‐1 inhibitor CH3 suppresses the growth of a broad range of cancers and both inhibitors overcome cisplatin resistance in OC. Thus, our findings suggest that targeting And‐1 by its inhibitors is a potential broad‐spectrum anti‐cancer chemotherapy regimen.

## CONFLICT OF INTERESTS

The authors declare that there is no conflict of interest that could be perceived as prejudicing the impartiality of the research reported.

## Supporting information

Table S1‐S3

Supporting Information

## Data Availability

The data that supports the findings of this study are available in the supplementary material of this article. The detailed data of NCI60 cell screening are available from the corresponding author upon reasonable request. These data will be available for public in three years based on NCI/DTP policy.
