# Supplementary material for: Discovery and characterization of potent And‐1 inhibitors for cancer treatment
Source: Clin Transl Med. 2021 Dec 19;11(12):e627. doi: 10.1002/ctm2.627 (PMC8684776; doi:10.1002/ctm2.627)
Supplement: Supplementary file 1 — Table S1‐S3 [file CTM2-11-e627-s001.docx]

**Table S1. Identified compounds that reduce luciferase activity by qHTS assay**

| **Sample Name** | **IC50 (uM) 1** | **IC50 (uM) 2** | **IC50 (uM) average** |
| --- | --- | --- | --- |
| Methotrexate hydrate | 0.08 | 0.16 | 0.12 |
| Diphenyleneiodonium chloride | 0.84 | 0.03 | 0.43 |
| AC-93253 iodide | 1.09 | 0.44 | 0.76 |
| PD-166285 hydrate | 3.33 | 2.73 | 3.03 |
| Thapsigargin | 13.27 | 0.63 | 6.95 |
| Calcimycin | 6.10 | 10.54 | 8.32 |
| NSC 95397 | 11.17 | 7.68 | 9.42 |
| Resveratrol | 14.89 | 22.93 | 18.91 |
| Cilnidipine | 22.93 | 19.30 | 21.11 |
| PAC-1 | 18.75 | 23.83 | 21.29 |
| Gemcitabine hydrochloride | 0.02 | inactive |  |
| (S)-(+)-Camptothecin | 0.73 | inactive |  |
| 2-methoxyestradiol | 1.67 | inactive |  |
| Topotecan hydrochloride hydrate | 0.61 | inactive |  |

**Table S2. Clinical characteristics of 11 ovarian cancer patients**

| Patient | Histology | Grade | Chemotherapy | TFI |
| --- | --- | --- | --- | --- |
| 1 | Serous | 1 | Carboplatin + Taxol | 4 |
| 2 | Serous | 1 | Carboplatin + Taxol | 1 |
| 3 | Serous | 1 | Carboplatin + Taxol | 4 |
| 4 | Serous | 3 | Cisplatin + CTX +VP-16 | 4 |
| 5 | Serous | 1 | Carboplatin + Taxol+ Pazopanib | 5 |
| 6 | Serous | 2 | Carboplatin + Taxol | 4 |
| 7 | Serous | 2 | Carboplatin + Taxol | 5 |
| 8 | Serous | 3 | Carboplatin + Taxol + Pazopanib | 5 |
| 9 | Serous | 2 | Cisplatin + Taxol | 2 |
| 10 | Serous | 2 | Carboplatin + Taxol | 1 |
| 11 | Serous | 1 | Carboplatin + Taxol | 4 |

**Table S3. List of And-1 (WDHD1)**-**ATR signature genes**

| WDHD1 | ATR | ATRIP | CHEK1 | \| MCM2 \| \| --- \| |
| --- | --- | --- | --- | --- | --- |
| CHEK2 | CDC6 | RPA2 | WRN | CDC45 |
| MCM7 | MCM6 | MCM5 | MCM4 | MCM3 |
| GINS | BRCA1 | RAD51 | MRE11 | NBS1 |
| CTIP | BRCA2 | RAD50 |  |  |
