# Supplementary material for: Discovery and characterization of potent And‐1 inhibitors for cancer treatment
Source: Clin Transl Med. 2021 Dec 19;11(12):e627. doi: 10.1002/ctm2.627 (PMC8684776; doi:10.1002/ctm2.627)
Supplement: Supplementary file 2 — Supporting Information [file CTM2-11-e627-s002.pdf]

Supplementary data

Discovery and characterization of potent And-1 inhibitors for cancer treatment

Jing Li, Yi Zhang, Jing Sun, Leyuan Chen, Wenfeng Gou, Chi-Wei Chen, Yuan Zhou, Zhuqing Li, David W. Chan, Ruili Huang, Huadong Pei, Wei Zheng, Yiliang Li, Menghang Xia, Wenge Zhu

This file includes:

1. Supplementary Fig. S1-S5

2. Supplementary Table. S1-S3

3. Supplementary materials and methods

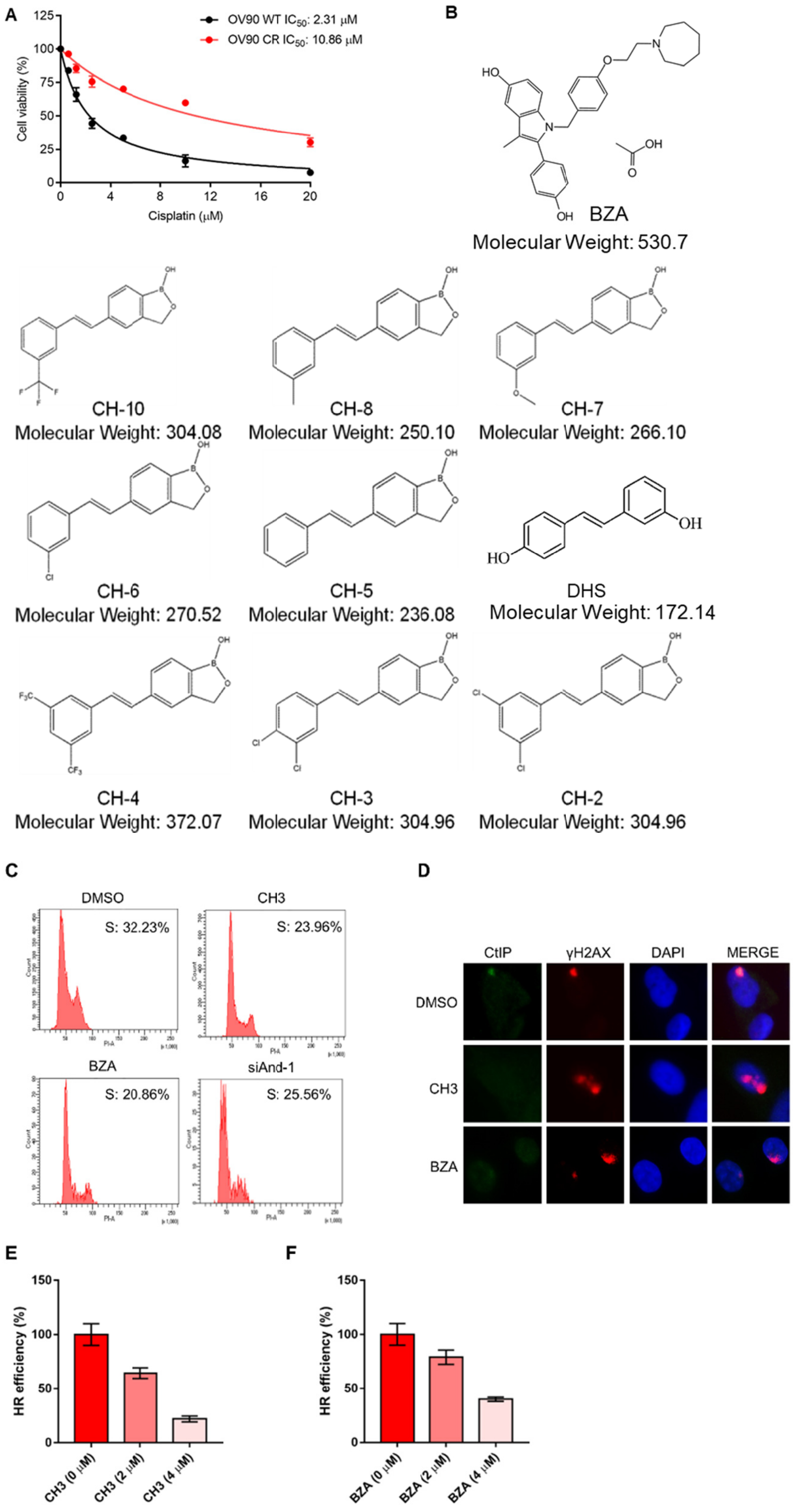
A
OV90 WT IC50: 2.31 μM
OV90 CR IC50: 10.86 μM
Cell viability (%)
Cisplatin (μM)
B
BZA
Molecular Weight: 530.7
CH-10
Molecular Weight: 304.08
CH-8
Molecular Weight: 250.10
CH-7
Molecular Weight: 266.10
CH-6
Molecular Weight: 270.52
CH-5
Molecular Weight: 236.08
DHS
Molecular Weight: 172.14
CH-4
Molecular Weight: 372.07
CH-3
Molecular Weight: 304.96
CH-2
Molecular Weight: 304.96
C
DMSO
S: 32.23%
CH3
S: 23.96%
BZA
S: 20.86%
siAnd-1
S: 25.56%
D
CtIP
γH2AX
DAPI
MERGE
DMSO
CH3
BZA
E
HR efficiency (%)
CH3 (0 μM)
CH3 (2 μM)
CH3 (4 μM)
F
HR efficiency (%)
BZA (0 μM)
BZA (2 μM)
BZA (4 μM)

Supplementary Figure S1. Identification of novel And-1 inhibitors. (A) SRB assay to detect cell viability of OV90 WT and OV90 CR cells treated with cisplatin at indicated concentrations. (**B)** Chemical structures of resveratrol analogs. Molecular weight of each compound was indicated. (**C)** Flow cytometry analysis of propidium-iodide stained IGROV1 cells treated with DMSO, 1 μM of CH3, 1 μM of BZA or siAnd-1. S: S phase. (**D)** Immunofluorescence assay to detect CtIP and γH2AX foci formation after CH3 or BZA treatments in IGROV1 micro-irradiated to generate DSBs. IGROV1 cells treated with DMSO, 1 μM of CH3 or 1 μM of BZA were micro-irradiated and then cells were harvested 30 min post irradiation to immunostain γH2AX and CtIP. For each experiment, 50 cells were counted and the percentage of cells with co-localization of γH2AX and CtIP foci was determined. Data represent means ± SEM from three independent experiments. (**E**) HR reporter assay to detect HR repair efficiency in cells treated with CH3. IGROV1 cells treated with DMSO, 2 μM or 4 μM of CH3 were subjected to HR reporter assay and the percentage of GFP-positive cells was determined by flow cytometry 48 h after I-SceI plasmid transfection. HR efficiency were normalized to those obtained from cells treated with DMSO. Data represent means ± SEM from three independent experiments. (**F**) HR reporter assay to detect HR repair efficiency in cells treated with BZA using approaches as in E.

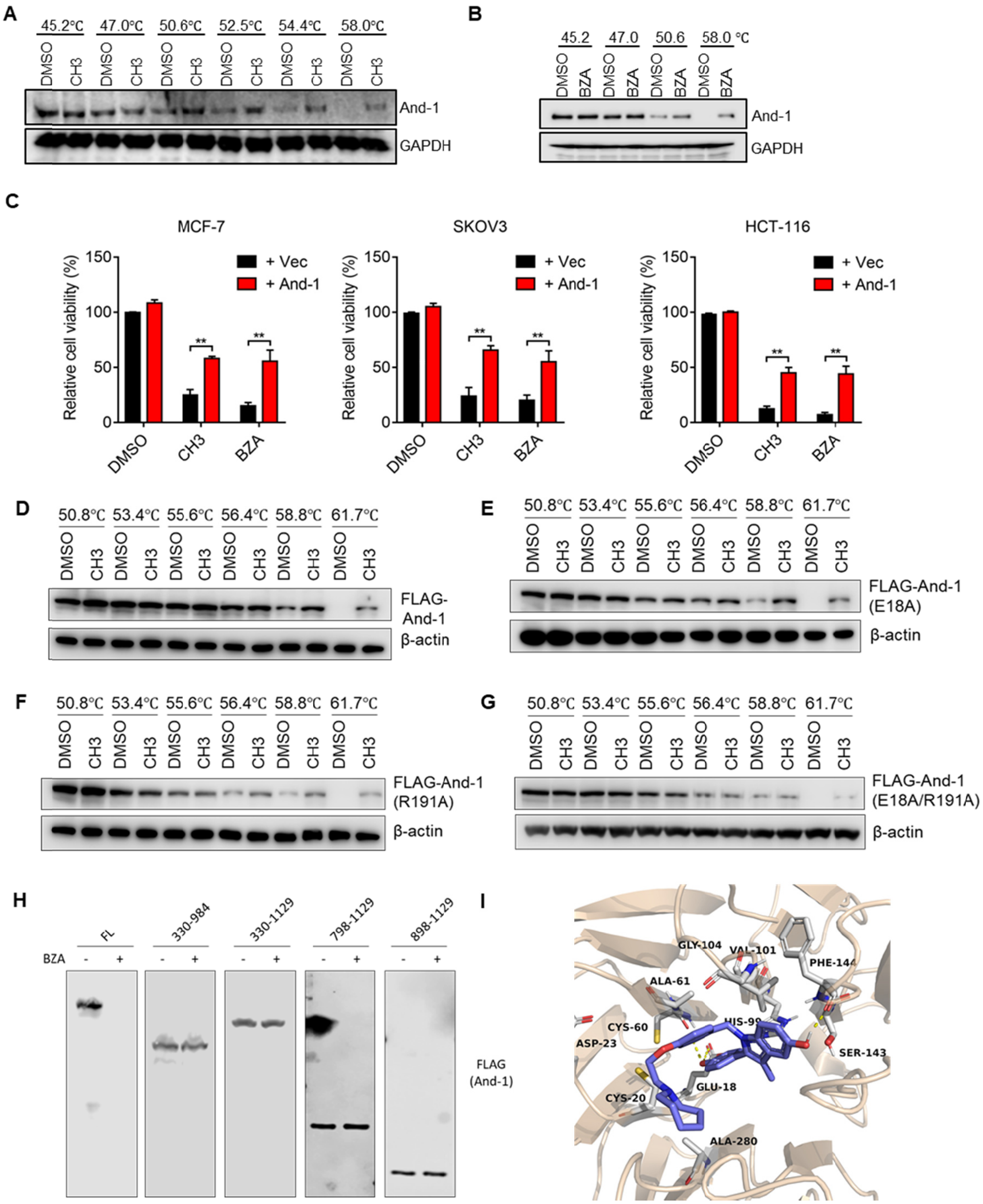

Supplementary Figure S2. And-1 inhibitors directly interact with And-1. (A-B) Thermal stability of And-1 in IGROV1 cells treated with CH3 (**A**) or BZA (**B**). (**C**) SRB assay to detect cell viability to indicated compounds in indicated cells transfected with overexpressed And-1 or vector control. (**D-G)** Thermal stability of FLAG-And-1-WT (**D),** FLAG-And-1-E18A (**E**), FLAG-And-1-R191A (**F**) and FLAG-And-1-E18A/R191A (**G**) in IGROV1 cells treated with CH3. (**H**) Immunoblot analyses to detect the expression of full-length FLAG-And-1 and its truncation mutants in cells treated with or without BZA. (**I**) Docking pose of BZA (blue) in WD40 domain of And-1 (yellow).

A

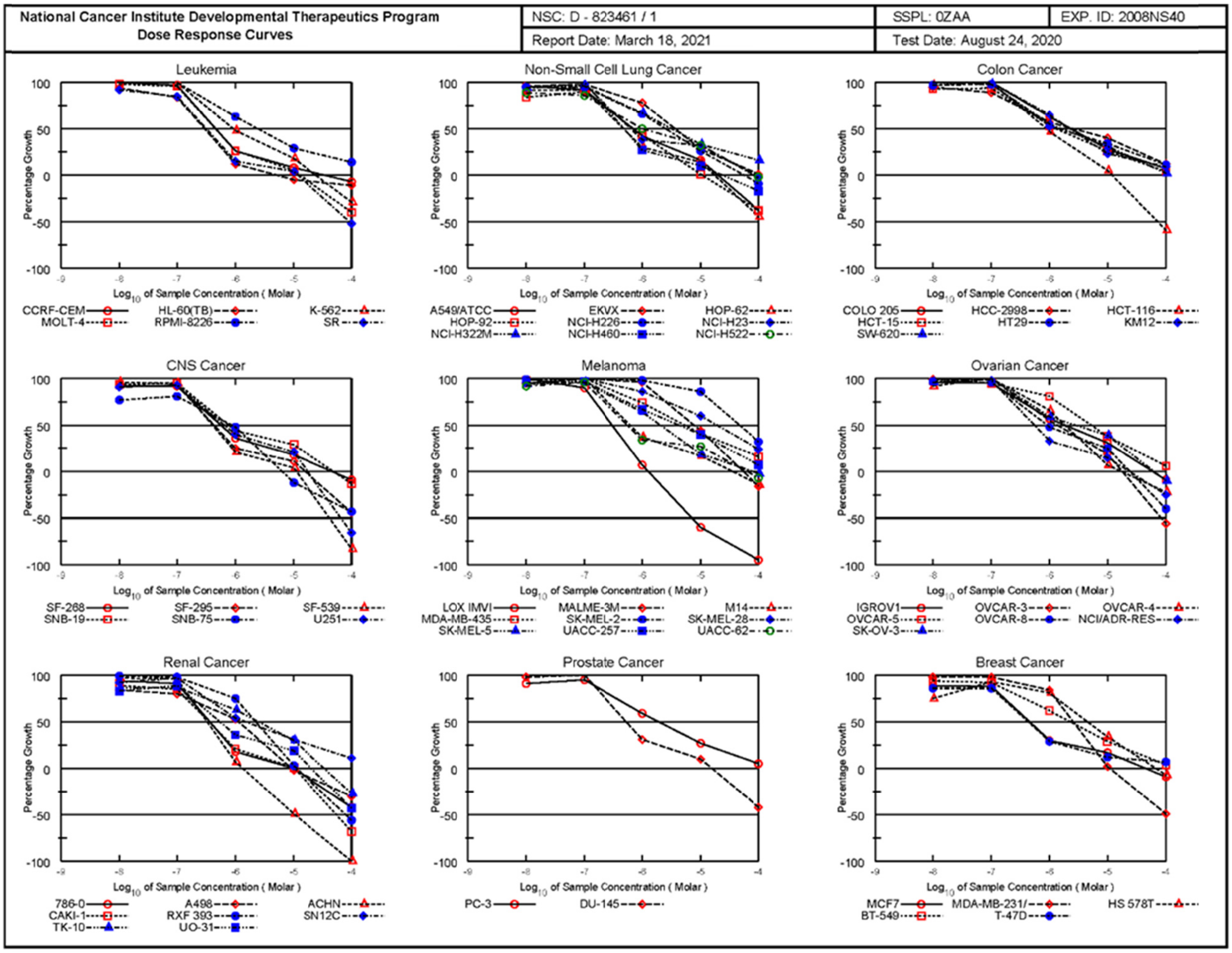

National Cancer Institute Developmental Therapeutics Program
Dose Response Curves
NSC: D - 823461 / 1
SSPL: 0ZAA
EXP. ID: 2008NS40
Report Date: March 18, 2021
Test Date: August 24, 2020
Leukemia
Non-Small Cell Lung Cancer
Colon Cancer
CNS Cancer
Melanoma
Ovarian Cancer
Renal Cancer
Prostate Cancer
Breast Cancer
Percentage Growth
Log10 of Sample Concentration (Molar)
CCRF-CEM HL-60(TB) K-562 MOLT-4 RPMI-8226 SR
A549/ATCC EKVX HOP-62 HOP-92 NCI-H226 NCI-H23 NCI-H322M NCI-H460 NCI-H522
COLO 205 HCC-2998 HCT-116 HCT-15 HT29 KM12 SW-620
SF-268 SF-295 SF-539 SNB-19 SNB-75 U251
LOX IMVI MALME-3M M14 MDA-MB-435 SK-MEL-2 SK-MEL-28 SK-MEL-5 UACC-257 UACC-62
IGROV1 OVCAR-3 OVCAR-4 OVCAR-5 OVCAR-8 NCI/ADR-RES SK-OV-3
786-0 A498 ACHN CAKI-1 RXF 393 SN12C TK-10 UO-31
PC-3 DU-145
MCF7 MDA-MB-231/ HS 578T BT-549 T-47D

B

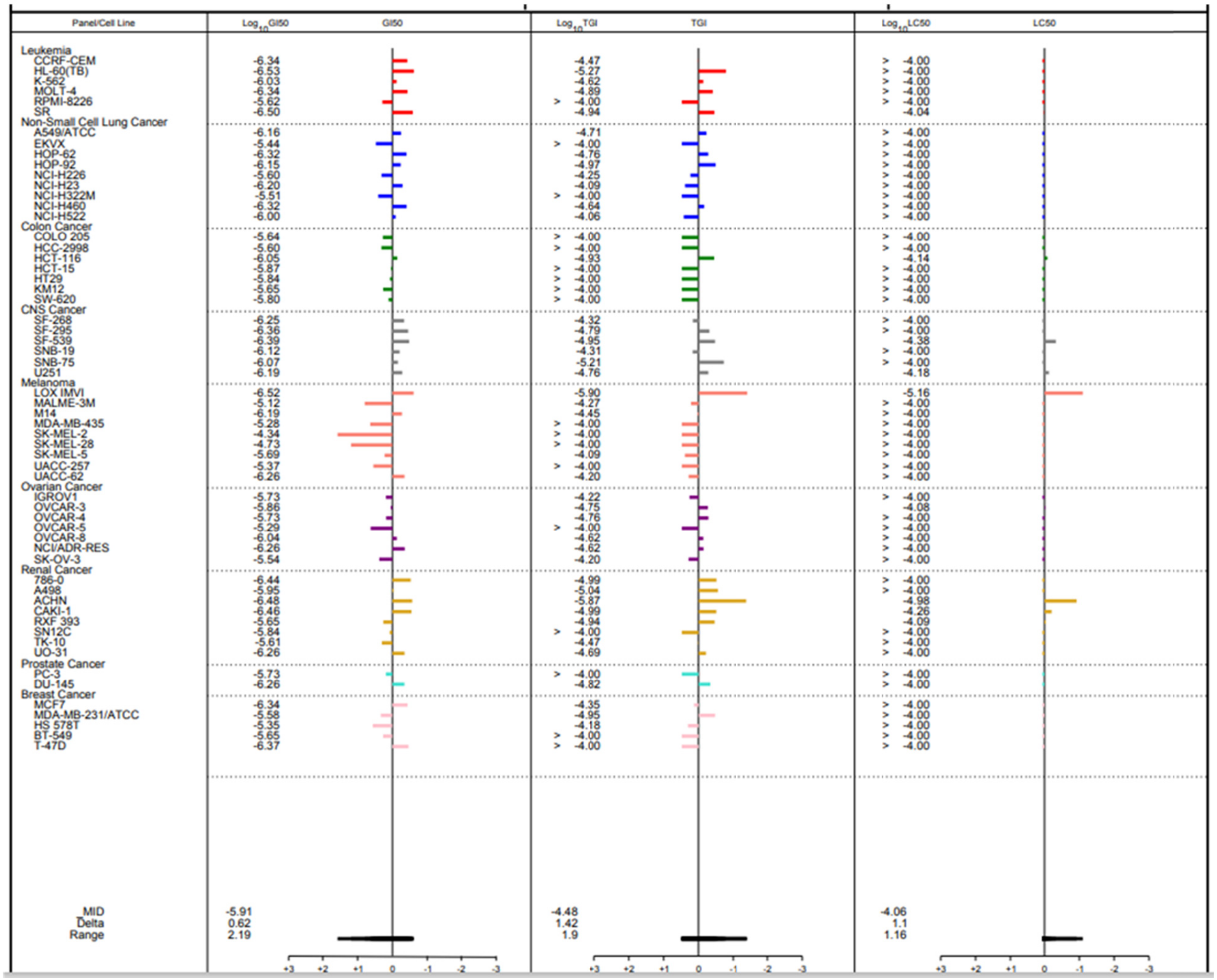

Supplementary Figure S3. CH3 inhibits cancer cell survival and tumor growth. (A) Growth inhibition of NCI60 cell lines after exposure to CH3 at following concentrations: 0.01 µM, 0.1 µM, 1 µM, 10 µM and 100 µM. **(B)** NCI-DTP 5-dose assay mean graphs. The positive and negative values are plotted along a vertical line that represents the mean response of all the cell lines to CH3. Positive values indicate that the cellular sensitivities to CH3 are less than average value, and negative values indicate that cellular sensitivities to CH3 exceed the mean. The concentration causing 50% cell growth inhibition (GI50), total cell growth inhibition (TGI), and 50% cell death (LC50) compared with the control.

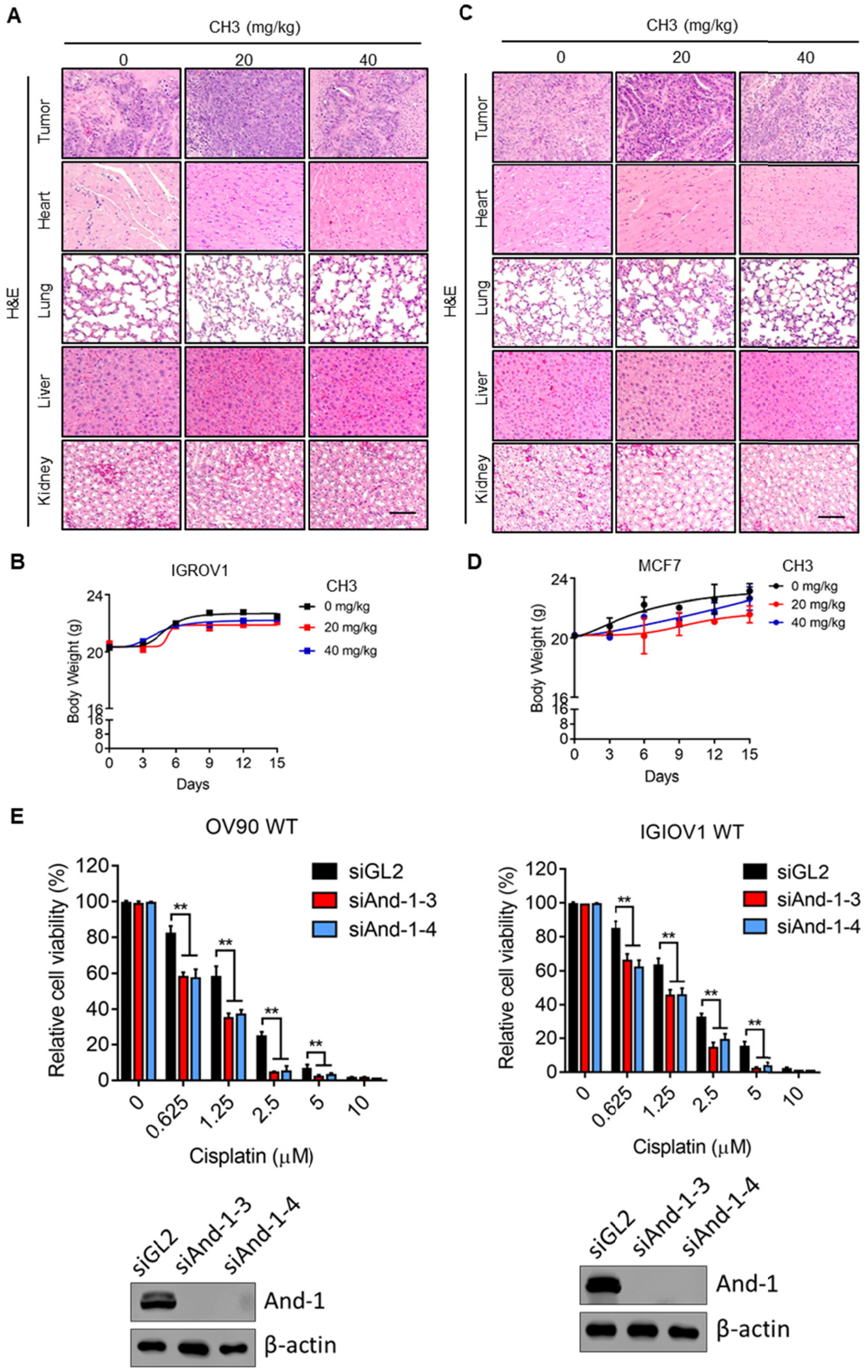

Supplementary Figure S4. CH3 inhibits tumor growth *in vivo*. (A) H&E staining of paraffin-embedded 3-μm-thick tissue sections of the tumor, heart, lung, liver and kidney in mice treated as in Figure 6A. (**B)** Body weight changes of mice in each group treated in Figure 6A. Data are represented as means ± SEM, n>=7 tumors/group. **(C)** H&E staining of paraffin-embedded 3-μm-thick tissue sections of the tumor, heart, lung, liver and kidney in mice treated as in Figure 6D. (**D)** Body weight changes of mice in each group treated in Figure 6D. Data are represented as means ± SEM, n>=7 tumors/group. (**E)** SRB assay to detect cell viability to cisplatin in indicated parental cells treated with indicated siRNAs. Lower panel, Western blotting for indicated proteins in cells treated as above.

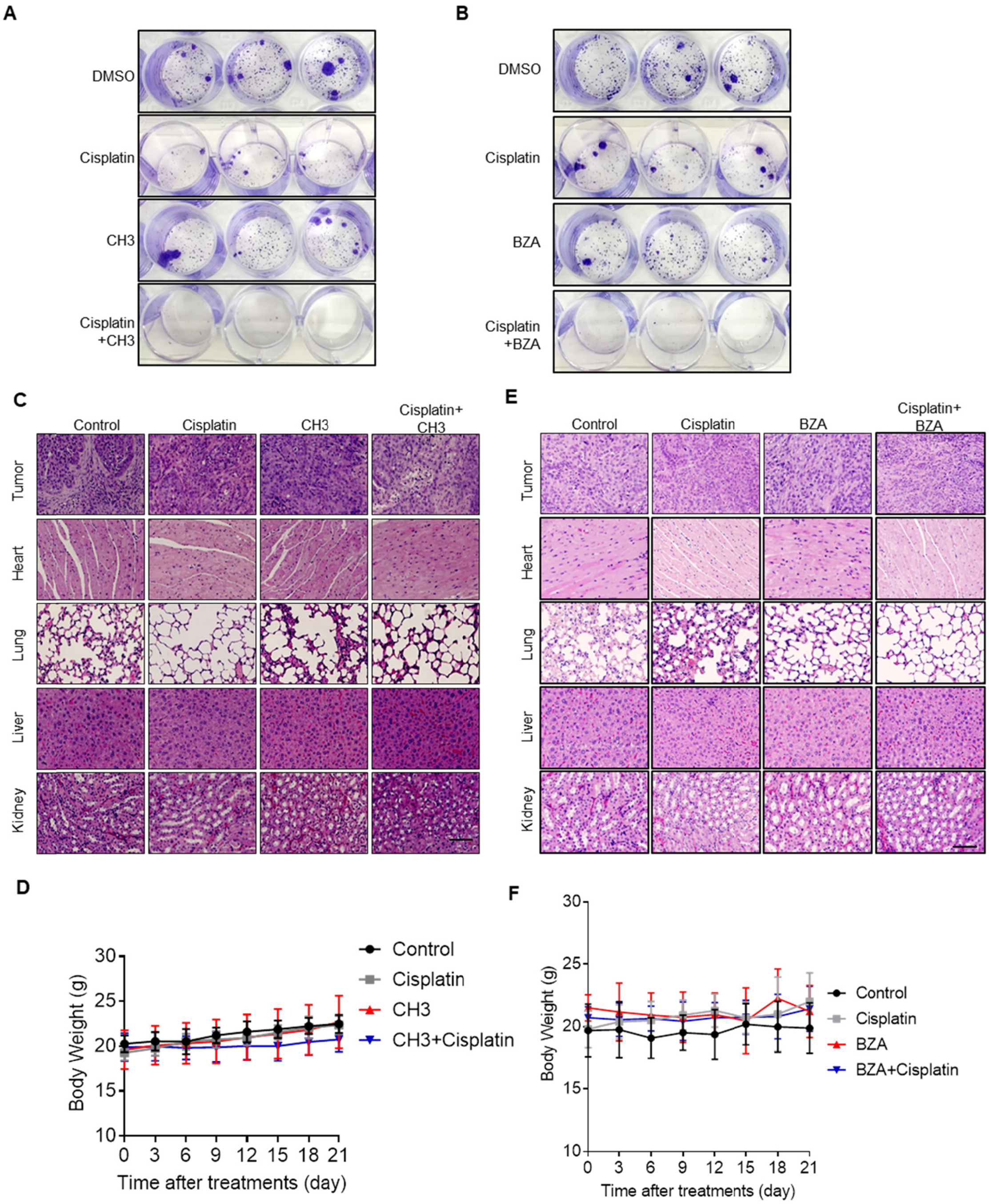

Supplementary Fig. S5. (A) Images of **c**olony formation in IGROV1 CR cells treated with DMSO, cisplatin, CH3 or combination of cisplatin and CH3. (**B**) Images of **c**olony formation in IGROV1 CR cells treated with DMSO, cisplatin, BZA or combination of cisplatin and BZA. (**C**) H&E staining of paraffin-embedded 3-µm-thick tissue sections of the tumor, heart, lung, liver and kidney in mice treated as in Figure 8C. (**D**) Body weight changes of mice in each group treated in Figure 8C. Data are represented as means ± SEM, n>=7 tumors/group. (**E**) H&E staining of paraffin-embedded 3-µm-thick tissue sections of the tumor, heart, lung, liver and kidney in mice treated as in Figure 8F. (**F**) Body weight changes of mice in each group treated in Figure 8F. Data are represented as means ± SEM, n>=7 tumors/group.

Table S1. Identified compounds that reduce luciferase activity by qHTS assay

Sample Name	IC50 (uM) 1	IC50 (uM) 2	IC50 (uM) average
Methotrexate hydrate	0.08	0.16	0.12
Diphenyleneiodonium chloride	0.84	0.03	0.43
AC-93253 iodide	1.09	0.44	0.76
PD-166285 hydrate	3.33	2.73	3.03
Thapsigargin	13.27	0.63	6.95
Calcimycin	6.10	10.54	8.32
NSC 95397	11.17	7.68	9.42
Resveratrol	14.89	22.93	18.91
Cilnidipine	22.93	19.30	21.11
PAC-1	18.75	23.83	21.29
Gemcitabine hydrochloride	0.02	inactive	
(S)-(+)-Camptothecin	0.73	inactive	
2-methoxyestradiol	1.67	inactive	
Topotecan hydrochloride hydrate	0.61	inactive	

Table S2. Clinical characteristics of 11 ovarian cancer patients

Patient	Histology	Grade	Chemotherapy	TFI
1	Serous	1	Carboplatin + Taxol	4
2	Serous	1	Carboplatin + Taxol	1
3	Serous	1	Carboplatin + Taxol	4
4	Serous	3	Cisplatin + CTX +VP-16	4
5	Serous	1	Carboplatin + Taxol+ Pazopanib	5
6	Serous	2	Carboplatin + Taxol	4
7	Serous	2	Carboplatin + Taxol	5
8	Serous	3	Carboplatin + Taxol + Pazopanib	5
9	Serous	2	Cisplatin + Taxol	2
10	Serous	2	Carboplatin + Taxol	1
11	Serous	1	Carboplatin + Taxol	4

Table S3. List of And-1 (WDHD1)-ATR signature genes

WDHD1	ATR	ATRIP	CHEK1	MCM2
CHEK2	CDC6	RPA2	WRN	CDC45
MCM7	MCM6	MCM5	MCM4	MCM3
GINS	BRCA1	RAD51	MRE11	NBS1
CTIP	BRCA2	RAD50		

3. Materials and Methods

3.1 Animal experiments

Mouse housing and all procedures were followed regulation accordance with protocols approved by the Institutional Animal Care and Use Committees (IACUC) of The George Washington University. 5-6-week old female BALB/c athymic nude mice (CByJ.Cg-Foxn1nu/J, weighting 20-25g, from the Jackson Laboratory) were inoculated subcutaneously by injecting the OC cells, cisplatin resistant OC cells or breast cancer cells suspended in 100 µl ice-cold Matrigel/PBS (1:1, V:V; 5 x 10^6/mouse) into the dorsal flank of each mouse. When the average tumor volume reached 100-150 mm^3, the mice were randomized into subsequent experiment groups. For tumors treated with CH3 only, CH3 was given at 0 mg/kg, 20 mg/kg and 40 mg/kg twice a week for three weeks. For combinational treatment, cisplatin, CH3 and BZA were then given intraperitoneally at 8 mg/kg, 20 mg/kg and 2 mg/kg respectively twice a week for three weeks. The relative tumor volumes were calculated using the formula: $a \times (b^2) / 2$, for which a and b represent the longest and shortest diameters, respectively. During the drug treatment, the tumor volume and body weight were measured. Tumor samples were collected 4 days after final administration of the drug.

3.2 Ovarian cancer patients

The specimens of chemosensitive and matched recurrent or chemoresistant tumor tissues from OC patients were kept as formalin-fixed paraffin-embedded (FFPE) samples in the Department of Pathology at The University of Hong Kong. Studies using human tissues were approved by the local institutional ethics committee (institutional review board reference No: UW 05-143 T/806 and UW 11-298). Written informed consent was received from patients prior to their inclusion in the study. The histological types, disease stages, and cancer cell contents in each FFPE section were examined by the three independent pathologists. Platinum-sensitive was defined as patients who have a total response to platinum-based therapy and no recurrence within 6 months. Platinum resistance means patients who had the recurrence occur within 6 months following the completion of platinum-based therapy.

3.3 Identification of And-1-ATR signature gene and analyses

The detailed approach to identify And-1-ATR signature gene (23 genes were identified) was described as below. The Kaplan Meier plotter is capable to assess the effect of 54k genes (mRNA, miRNA, protein) on survival in 21 cancer types including breast (n=7,830), ovarian (n=2,190), lung (n=3,452), and gastric (n=1,440) cancer. Sources for the databases include GEO, EGA, and TCGA. Primary purpose of the tool is a meta-analysis based discovery and validation of survival biomarkers. (Reference: Győrffy B. Survival analysis across the entire transcriptome identifies biomarkers with the highest prognostic power in breast cancer, Computational and Structural Biotechnology Journal, 2021; 19:4101-4109).

STEP1: Find top 100 gene of WDHD1-related gene by "Functional relationship" through PathwayNet

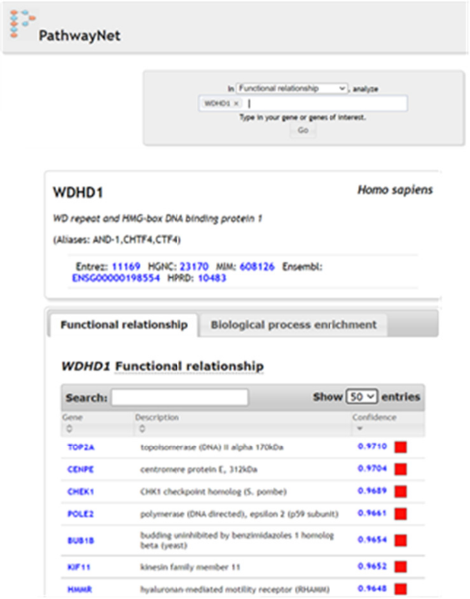

STEP2: Find top 100 gene of ATR-related gene by "Functional relationship" through PathwayNet

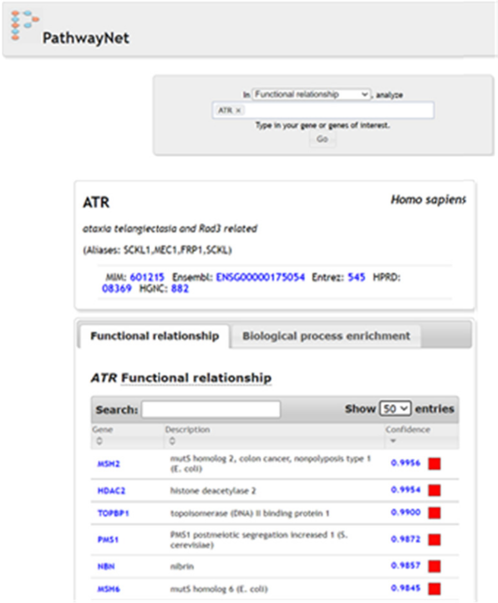

STEP3: Pick up most important and overlap genes from WDHD1 and ATR gene lists

List of WDHD1-ATR signature genes (23 genes)

WDHD1	ATR	ATRIP	CHEK1	MCM2
CHEK2	CDC6	RPA2	WRN	CDC45
MCM7	MCM6	MCM5	MCM4	MCM3
GINS	BRCA1	RAD51	MRE11	NBS1
CTIP	BRCA2	RAD50		

STEP4: Use And-1 (WDHD1) and ATR signature genes as a bait to check the PFS and OS in ovarian cancer patient through Km plotter

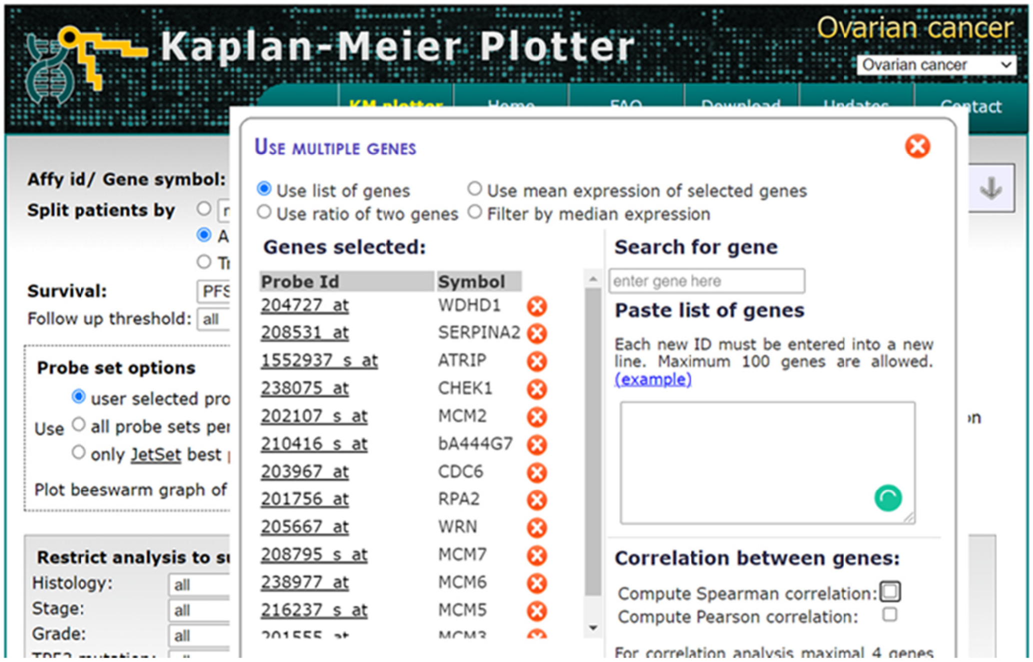

3.4 Synthesis and characterization of resveratrol derivatives.

The synthetic route of the compounds is shown in **Scheme 1**. The purity of the target compounds determined by high performance liquid chromatography were all higher than 95%. The structure of the intermediates and target compounds have been verified by ^{1}H-NMR and ^{13}C-NMR.

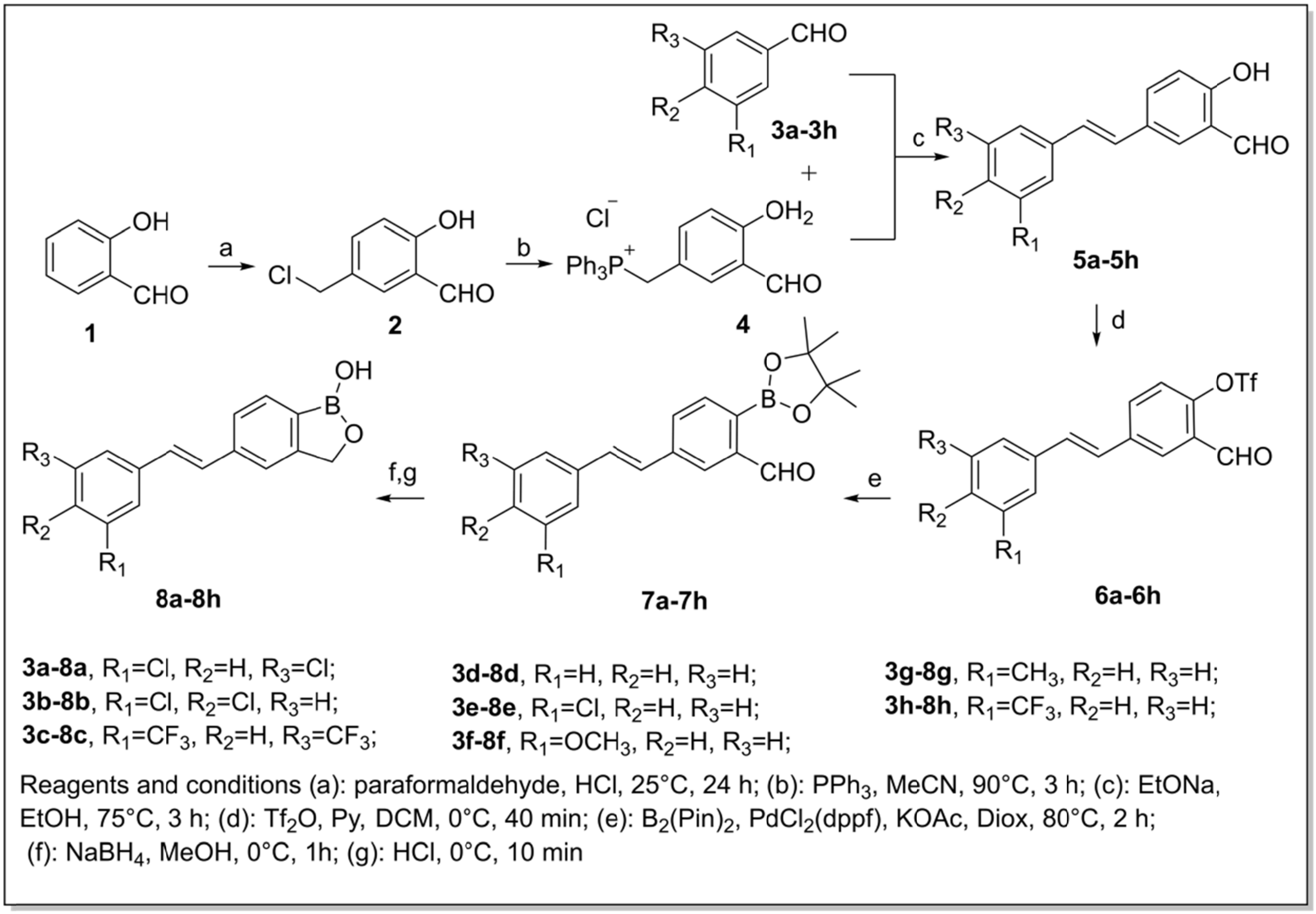

Scheme 1. The synthetic route of the compounds

Synthesis of 5-(chloromethyl)-2-hydroxybenzaldehyde (2)

A solution of paraformaldehyde (24.40 g, 0.20 mol) in concentrated hydrochloric acid (80 mL) was stirred at room temperature for 10 minutes, and then salicylaldehyde (17.95 g, 0.60 mol) was added dropwise over 30 minutes. The reaction solution was stirred at room temperature for 24 hours to give a white solid. Filtration, washing the filter cake with cold water, and drying to obtain a crude product. Recrystallization from n-hexane (310 mL) gave white crystals (16.80 g, 49.4%). ^{1}H NMR (400 MHz, DMSO-d_6) δ 10.91 (s, 1H), 10.27 (s, 1H), 7.72 (d, J = 2.4 Hz, 1H), 7.59 (dd, J = 8.5, 2.4 Hz, 1H), 7.03 (d, J = 8.5 Hz, 1H), 4.76 (s, 2H).

Synthesis of (3-formyl-4-hydroxybenzyl)triphenylphosphonium chloride (4)

5-(chloromethyl)-2-hydroxybenzaldehyde (15.50 g, 91.2 mmol) and triphenylphosphine (30.98 g, 118.1 mmol) were dissolved in 220 mL of dry acetonitrile. The reaction solution was stirred at 80°C for 3 hours under a nitrogen atmosphere. The acetonitrile was evaporated to dryness under reduced pressure, and the residue was washed with petroleum ether (100 mL×3) to afford white powder (38.52 g, 97.8%).

Synthesis of (E)-5-(3,5-dichlorostyryl)-2-hydroxybenzaldehyde (5a)

Sodium (0.19 g, 8.26 mmol) was dissolved in absolute ethanol (30 mL) under nitrogen. The phosphonium salt (1.20 g, 2.78 mmol) was added to this sodium ethoxide solution until the reaction mixture became dark yellow, and 3,5-dichlorobenzaldehyde (0.57 g, 3.26 mmol) was added. The reaction solution was stirred at 75°C for 3 hours. The reaction was withdrawn, cooled to room temperature, clarified and then turbid with water, and the pH was adjusted to be acidic with dilute hydrochloric acid to precipitate a yellow solid. Filtration, drying and recrystallization from tetrahydrofuran afforded yellow crystals (0.387 g, 44.4%). ^{1}H NMR (400 MHz, DMSO-d_6) δ 10.97 (s, 1H), 10.31 (s, 1H), 7.87 (d, *J* = 2.2 Hz, 1H), 7.79 (dd, *J* = 8.7, 2.3 Hz, 1H), 7.67 (d, *J* = 1.8 Hz, 2H), 7.46 (d, *J* = 16.5 Hz, 1H), 7.45 (t, *J* = 1.8 Hz, 1H), 7.13 (d, *J* = 16.5 Hz, 1H), 7.06 (d, *J* = 8.6 Hz, 1H).

By performing the steps described above, the following intermediates **5b-5h** were prepared:

(E)-5-(3,4-dichlorostyryl)-2-hydroxybenzaldehyde (*5b*), ^{1}H NMR (400 MHz, DMSO-d_6) δ 10.92 (s, 1H), 10.31 (s, 1H), 7.87 (d, *J* = 1.9 Hz, 2H), 7.80 (dd, *J* = 8.6, 2.3 Hz, 1H), 7.65 – 7.52 (m, 2H), 7.37 (d, *J* = 16.5 Hz, 1H), 7.13 (d, *J* = 16.5 Hz, 1H), 7.05 (d, *J* = 8.6 Hz, 1H). ^{13}C NMR (101 MHz, DMSO-d_6) δ 191.43, 161.22, 138.63, 134.57, 131.94, 131.13, 130.08, 129.72, 128.62, 128.24, 127.64, 126.74, 124.79, 122.88, 118.30;

(E)-5-(3,5-bis(trifluoromethyl)styryl)-2-hydroxybenzaldehyde (*5c*), ^{1}H NMR (400 MHz, DMSO-d_6) δ 11.02 (s, 1H), 10.32 (s, 1H), 8.29 (s, 2H), 7.93 (d, *J* = 2.0 Hz, 1H), 7.91 (s, 1H), 7.83 (dd, *J* = 8.6, 2.1 Hz, 1H), 7.67 (d, *J* = 16.6 Hz, 1H), 7.37 (d, *J* = 16.5 Hz, 1H), 7.07 (d, *J* = 8.6 Hz, 1H);

(E)-2-hydroxy-5-styrylbenzaldehyde (*5d*), ^{1}H NMR (400 MHz, DMSO-d_6) δ 10.88 (s, 1H), 10.31 (s, 1H), 7.86 (s, 1H), 7.84 – 7.78 (m, 1H), 7.59 (d, *J* = 7.7 Hz, 2H), 7.37 (t, *J* = 7.6 Hz, 2H), 7.30 – 7.20 (m, 2H), 7.14 (d, *J* = 16.5 Hz, 1H), 7.04 (d, *J* = 8.6 Hz, 1H);

***(E)-5-(3-chlorostyryl)-2-hydroxybenzaldehyde (*5e), ^{1}H NMR (400 MHz, DMSO-d_6) δ 10.94 (s, 1H), 10.31 (s, 1H), 7.87 (d, J = 2.0 Hz, 1H), 7.81 (dd, J = 8.6, 2.1 Hz, 1H), 7.68 (s, 1H), 7.54 (d, J = 7.8 Hz, 1H), 7.35 (dt, J = 17.0, 7.9 Hz, 3H), 7.14 (d, J = 16.5 Hz, 1H), 7.05 (d, J = 8.6 Hz, 1H);**

***(E)-2-hydroxy-5-(3-methoxystyryl)benzaldehyde (*5f),** ^{1}H NMR (400 MHz, DMSO-d_6) δ 10.89 (s, 1H), 10.31 (s, 1H), 7.93 – 7.75 (m, 2H), 7.39 – 7.22 (m, 2H), 7.14 (dd, J = 16.7, 12.2 Hz, 3H), 7.04 (d, J = 8.6 Hz, 1H), 6.88 – 6.79 (m, 1H), 3.79 (s, 3H);

***(E)-2-hydroxy-5-(3-methylstyryl)benzaldehyde (*5g),** ^{1}H NMR (400 MHz, DMSO-d_6) δ 10.86 (s, 1H), 10.30 (s, 1H), 7.84 (d, J = 2.2 Hz, 1H), 7.80 (dd, J = 8.6, 2.3 Hz, 1H), 7.44 – 7.34 (m, 2H), 7.29 – 7.17 (m, 2H), 7.14 – 7.01 (m, 3H), 2.32 (s, 3H);

***(E)-2-hydroxy-5-(3-(trifluoromethyl)styryl)benzaldehyde (*5h),** ^{1}H NMR (400 MHz, DMSO-d_6) δ 10.93 (s, 1H), 10.31 (s, 1H), 7.95 (s, 1H), 7.90 (t, J = 4.3 Hz, 2H), 7.83 (dd, J = 8.7, 2.3 Hz, 1H), 7.65 – 7.55 (m, 2H), 7.44 (d, J = 16.5 Hz, 1H), 7.26 (d, J = 16.5 Hz, 1H), 7.06 (d, J = 8.6 Hz, 1H);

Synthesis of (E)-4-(3,5-dichlorostyryl)-2-formylphenyl trifluoromethanesulfonate (6a)

5a (0.557 g, 1.90 mmol) and pyridine (0.750 g, 9.49 mmol) were dissolved in anhydrous dichloromethane (25 mL), and the mixture was stirred at 0°C. A solution of trifluoromethanesulfonic anhydride (0.810 g, 2.87 mmol) in dichloromethane was added dropwise. After the reaction solution was stirred for 40 minutes in an ice bath, it was monitored by thin layer chromatography (TLC) to determine when reactants were consumed and a new product was formed. The reaction mixture was poured into ice water, and the mixture was adjusted to pH 2 with dilute aqueous hydrochloric acid, washed with saturated brine, dried over anhydrous sodium sulfate and evaporated to dryness. The residue was purified by column chromatography (petroleum ether: ethyl acetate 4:1). A white solid (0.775 g, 96.3%) was obtained. ^{1}H NMR (400 MHz, DMSO-d_6) δ 10.11 (s, 1H), 8.28 (s, 1H), 8.03 (d, J = 8.6 Hz, 1H), 7.71 (s, 2H), 7.65 (d, J = 8.6 Hz, 1H), 7.60 (d, J = 16.5 Hz, 1H), 7.50 (s, 1H), 7.41 (d, J = 16.5 Hz, 1H). ^{13}C NMR (101 MHz, DMSO-d_6) δ 189.15, 147.29 , 140.62, 138.38, 134.99, 134.27, 131.74, 129.48, 129.21, 128.85, 127.78, 125.79, 123.84, 123.43, 120.24, 117.06, 113.87.

By performing the steps described in Example 4, the following intermediates were prepared:

(E)-4-(3,4-dichlorostyryl)-2-formylphenyl trifluoromethanesulfonate (6b)

^{1}H NMR (400 MHz, DMSO-d_6) δ 10.11 (s, 1H), 8.28 (d, J = 2.1 Hz, 1H), 8.02 (dd, J = 8.6, 2.2 Hz, 1H), 7.90 (d, J = 1.4 Hz, 1H), 7.68 – 7.56 (m, 3H), 7.51 (d, J = 16.5 Hz, 1H), 7.41 (d, J = 16.5 Hz, 1H). ^{13}C NMR (101 MHz, DMSO-d_6) δ 189.20, 147.11, 138.55, 137.70, 134.14, 132.10, 131.71, 131.33, 130.92, 129.79, 128.83, 128.79, 128.23, 127.42, 123.77, 120.24, 117.05;

(E)-4-(3,5-bis(trifluoromethyl)styryl)-2-formylphenyl-trifluoromethanesulfonate (6c)

^{1}H NMR (400 MHz, DMSO-d_6) δ 10.12 (s, 1H), 8.47 – 8.28 (m, 3H), 8.09 (dd, J = 8.6, 2.1 Hz, 1H), 8.03 (s, 1H), 7.84 (d, J = 16.6 Hz, 1H), 7.69 (dd, J = 12.3, 10.5 Hz, 2H);

(E)-2-formyl-4-styrylphenyl trifluoromethanesulfonate (6d)

^{1}H NMR (400 MHz, DMSO-d_6) δ 10.12 (s, 1H), 8.35 (d, J = 2.3 Hz, 1H), 8.07 (dd, J = 8.6, 2.3 Hz, 1H), 7.65 (dd, J = 14.2, 8.0 Hz, 3H), 7.53 – 7.39 (m, 4H), 7.34 (t, J = 7.3 Hz, 1H);

(E)-4-(3-chlorostyryl)-2-formylphenyl trifluoromethanesulfonate (6e)

^{1}H NMR (400 MHz, DMSO-d_6) δ 10.11 (s, 1H), 8.33 (d, J = 2.3 Hz, 1H), 8.06 (dd, J = 8.6, 2.3 Hz, 1H), 7.76 (s, 1H), 7.63 (dd, J = 18.5, 8.1 Hz, 2H), 7.53 (d, J = 16.5 Hz, 1H), 7.50 – 7.35 (m, 3H);

(E)-2-formyl-4-(3-methoxystyryl)phenyl trifluoromethanesulfonate (6f)

^{1}H NMR (400 MHz, DMSO-d_6) δ 10.11 (s, 1H), 8.34 (d, J = 1.9 Hz, 1H), 8.06 (dd, J = 8.6, 2.0 Hz, 1H), 7.64 (d, J = 8.5 Hz, 1H), 7.45 (s, 2H), 7.34 (t, J = 7.9 Hz, 1H), 7.23 (d, J = 7.3 Hz, 2H), 6.99 – 6.86 (m, 1H), 3.82 (s, 3H);

(E)-2-formyl-4-(3-methylstyryl)phenyl trifluoromethanesulfonate (6g)

^{1}H NMR (400 MHz, DMSO-d_6) δ 10.11 (s, 1H), 8.34 (d, J = 2.3 Hz, 1H), 8.06 (dd, J = 8.6, 2.3 Hz, 1H), 7.62 (d, J = 8.6 Hz, 1H), 7.52 – 7.39 (m, 4H), 7.31 (t, J = 7.6 Hz, 1H), 7.16 (d, J = 7.5 Hz, 1H), 2.35 (s, 3H);

(E)-2-formyl-4-(3-(trifluoromethyl)styryl)phenyl trifluoromethanesulfonate (6h)

^{1}H NMR (400 MHz, DMSO-d_6) δ 10.12 (s, 1H), 8.36 (d, J = 2.3 Hz, 1H), 8.09 (dd, J = 8.6, 2.3 Hz, 1H), 8.03 (s, 1H), 7.96 (d, J = 6.7 Hz,1H), 7.72 – 7.54 (m, 5H);

Synthesis of (E)-5-(3,5-dichlorostyryl)benzo[c][1,2]oxaborol-1(3H)-ol (8a)(CH2)

$B_2(Pin)_2$ (0.870 g, 3.43 mmol) and potassium acetate (0.510 g, 5.19 mmol) were added to dry 1,4-dioxane (18 mL) under nitrogen atmosphere. (E)-4-(3,5-dichlorostyryl)-2-formylphenyl trifluoromethanesulfonate (0.725 g, 1.71 mmol) and Pd(dppf)Cl_2 (0.140 g, 0.17 mmol) were then added. The mixture was stirred at 80°C for two hours. After the dioxane was removed under reduced pressure, the residue was purified by flash column chromatography to obtain a crude product, which was directly used in the next reaction. The crude product was dissolved in a mixed solution of 4 mL methanol and 3 mL tetrahydrofuran, and $NaBH_4$ (0.280 g, 7.41 mmol) was added in portions at 0°C. After the mixture was stirred for one hour, part of the solvent was removed under reduced pressure, 15 mL of water was added, and the pH was adjusted to 3 with dilute hydrochloric acid. After extraction with ethyl acetate, the organic phase was dried over anhydrous sodium sulfate and evaporated to dryness under reduced pressure. The residue was first purified by column chromatography and then recrystallized from toluene to obtain 0.250 g of white solid. The two-step yield was 48.2%. ^{1}H NMR (400 MHz, DMSO-d_6) δ 9.23 (s, 1H), 7.76 (d, J = 7.6 Hz, 1H), 7.72 (d, J = 1.7 Hz, 2H), 7.65 – 7.57 (m, 2H), 7.54 (d, J = 16.5 Hz, 1H), 7.50 (s, 1H), 7.33 (d, J = 16.4 Hz, 1H), 5.04 (s, 2H). ^{13}C NMR (101 MHz, DMSO-d_6) δ 155.02, 141.25, 139.06, 134.92, 132.22, 131.27, 127.20, 127.15, 126.26, 125.47, 119.81, 70.29.

By performing the steps described in above, the following intermediates were prepared:

(E)-5-(3,4-dichlorostyryl)benzo[c][1,2]oxaborol-1(3H)-ol (8b)(CH3)

^{1}H NMR (400 MHz, DMSO-d_6) δ 9.23 (s, 1H), 7.89 (s, 1H), 7.74 (d, J = 7.6 Hz, 1H), 7.65 – 7.54 (m, 4H), 7.43 (d, J = 16.5 Hz, 1H), 7.32 (d, J = 16.5 Hz, 1H), 5.02 (s, 2H). ^{13}C NMR (75 MHz, DMSO-d_6) δ 155.05, 139.25, 138.35, 132.02, 131.28, 130.27, 128.58, 127.46, 127.13, 126.18, 119.70, 70.28;

(E)-5-(3,5-bis(trifluoromethyl)styryl)benzo[c][1,2]oxaborol-1(3H)-ol (8c)(CH4)

^{1}H NMR (400 MHz, DMSO-d_6) δ 9.24 (s, 1H), 8.31 (s, 2H), 7.93 (s, 1H), 7.77 (d, J = 7.6 Hz, 1H), 7.71 (d, J = 16.5 Hz, 1H), 7.67 – 7.59 (m, 2H), 7.54 (d, J = 16.5 Hz, 1H), 5.03 (s, 2H). ^{13}C NMR (101 MHz, DMSO-d_6) δ 155.04, 140.32, 138.97, 133.09, 131.34, 131.17 (q, $^2J_{C-F}$ = 35.8 Hz), 127.31, 126.94, 126.34, 123.84 (q, $^1J_{C-F}$ = 273.9 Hz), 120.91, 119.90, 70.26;

(E)-5-styrylbenzo[c][1,2]oxaborol-1(3H)-ol (8d)(CH5)

^{1}H NMR (400 MHz, DMSO-d_6) δ 9.21 (s, 1H), 7.75 (d, J = 7.6 Hz, 1H), 7.60 (dd, J = 12.1, 7.7 Hz, 4H), 7.38 (t, J = 7.6 Hz, 2H), 7.35 – 7.24 (m, 3H), 5.01 (s, 2H). ^{13}C NMR (101 MHz, DMSO-d_6) δ 155.07, 139.78, 137.33, 131.25, 130.02, 129.21, 128.91, 128.34, 127.09, 125.98, 119.45, 70.31;

(E)-5-(3-chlorostyryl)benzo[c][1,2]oxaborol-1(3H)-ol (8e)(CH6)

^{1}H NMR (400 MHz, DMSO-d_6) δ 9.22 (s, 1H), 7.75 (d, J = 7.6 Hz, 1H), 7.71 (s, 1H), 7.59 (dd, J = 13.9, 6.6 Hz, 3H), 7.46 – 7.37 (m, 2H), 7.37 – 7.30 (m, 2H), 5.02 (s, 2H). ^{13}C NMR (101 MHz, DMSO-d_6) δ 155.04, 139.70, 139.40, 134.09, 131.26, 130.97, 130.62, 128.48, 127.92, 126.51, 126.14, 125.77, 119.65, 70.29;

(E)-5-(3-methoxystyryl)benzo[c][1,2]oxaborol-1(3H)-ol (8f)(CH7)

^{1}H NMR (400 MHz, DMSO-d_6) δ 9.21 (s, 1H), 7.74 (d, J = 7.6 Hz, 1H), 7.66 – 7.55 (m, 2H), 7.37 – 7.26 (m, 3H), 7.20 (d, J = 6.3 Hz, 2H), 6.87 (dd, J = 8.0, 1.8 Hz, 1H), 5.02 (s, 2H), 3.80 (s, 3H). ^{13}C NMR (101 MHz, DMSO-d_6) δ 160.08, 155.06, 139.75, 138.80, 131.24, 130.19, 129.97, 129.21, 126.00, 119.67, 119.46, 114.11, 112.19, 70.29, 55.53;

(E)-5-(3-methylstyryl)benzo[c][1,2]oxaborol-1(3H)-ol (8g)(CH8)

^{1}H NMR (400 MHz, DMSO-d_6) δ 9.20 (s, 1H), 7.74 (d, J = 7.6 Hz, 1H), 7.64 – 7.54 (m, 2H), 7.47 – 7.38 (m, 2H), 7.33 – 7.22 (m, 3H), 7.10 (d, J = 7.5 Hz, 1H), 5.02 (s, 2H), 2.33 (s, 3H). ^{13}C NMR (101 MHz, DMSO-d_6) δ 155.07, 139.84, 138.27, 137.25, 131.23, 130.09, 129.08, 128.71, 127.56, 125.95, 124.39, 119.39, 70.30, 21.48;

(E)-5-(3-(trifluoromethyl)styryl)benzo[c][1,2]oxaborol-1(3H)-ol (8h)(CH10)

^{1}H NMR (400 MHz, DMSO-d_6) δ 9.22 (s, 1H), 7.94 (dd, J = 12.7, 8.8 Hz, 2H), 7.76 (d, J = 7.6 Hz, 1H), 7.62 (dd, J = 14.5, 8.9 Hz, 4H), 7.51 (d, J = 16.5 Hz, 1H), 7.45 (d, J = 16.5 Hz, 1H), 5.03 (s, 2H). ^{13}C NMR (101 MHz, DMSO-d_6) δ 155.05, 139.36, 138.56, 131.27, 130.99, 130.72, 130.21, 130.11 (q, $^{2}J_{C\text{-}F}$ = 31.4 Hz), 128.42, 126.18, 124.69 (q, $^{1}J_{C\text{-}F}$ = 273.4 Hz), 124.53 (d, $^{3}J_{C\text{-}F}$ = 3.5 Hz), 123.48 (d, $^{3}J_{C\text{-}F}$ = 3.7 Hz), 119.71, 70.28;

The following data are the ^{1}H-NMR and ^{13}C-NMR spectrum of the compounds **8a-8h**.

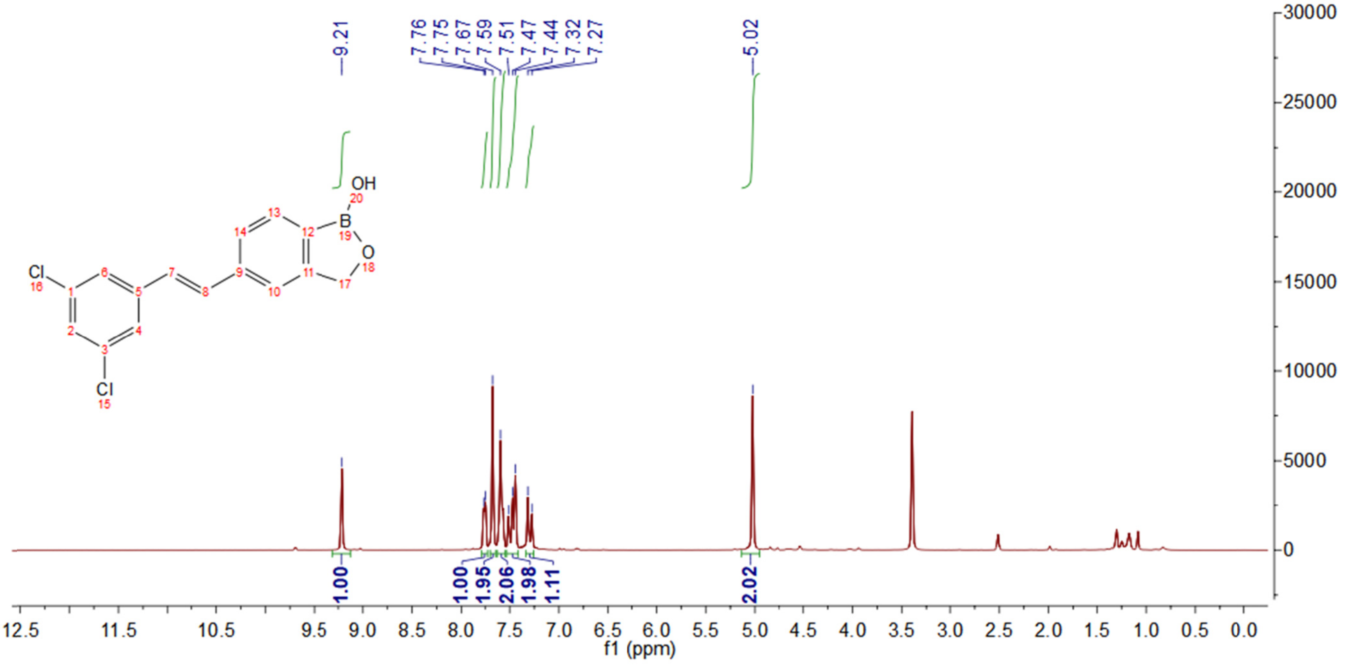

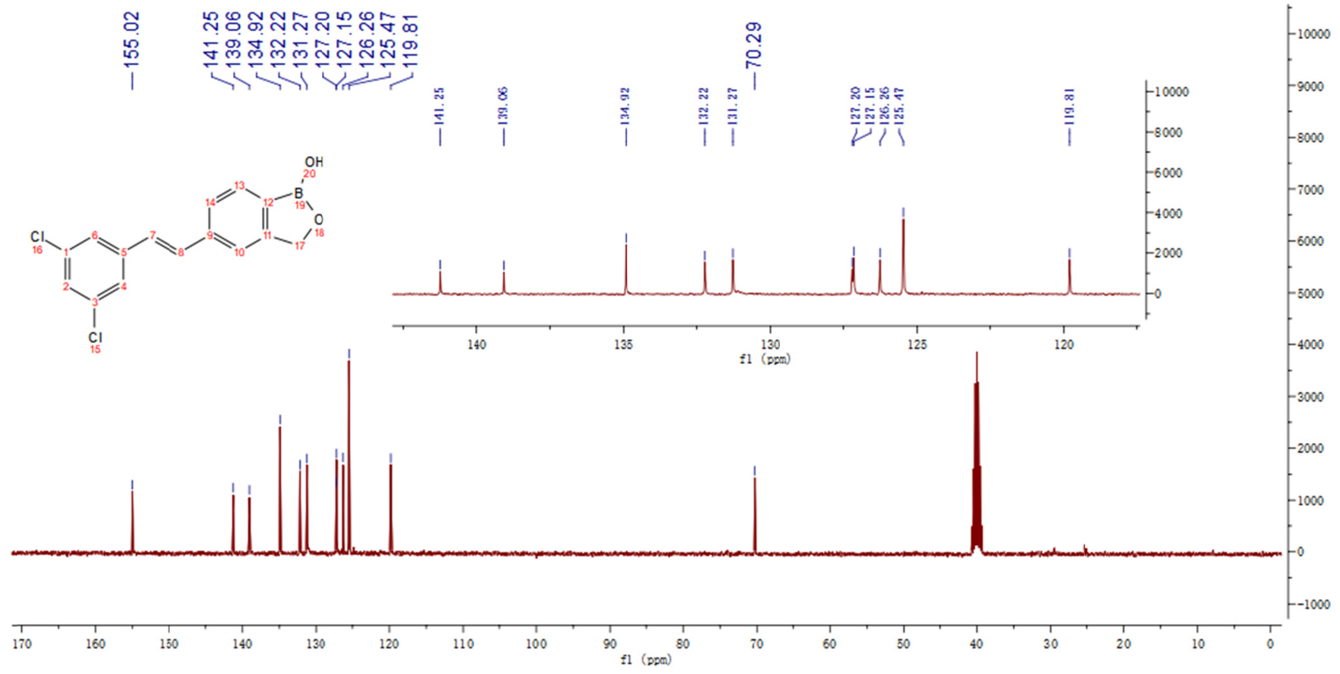

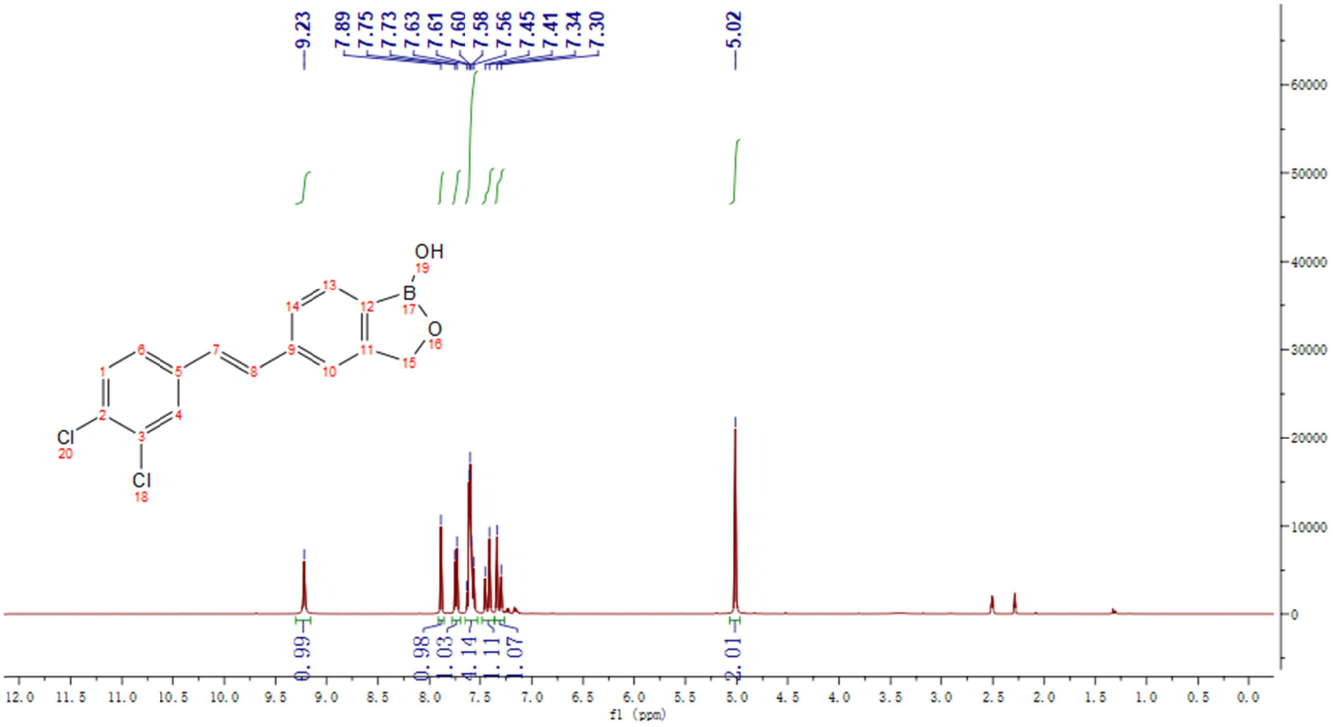
9.23
7.89
7.75
7.73
7.63
7.61
7.60
7.58
7.56
7.45
7.41
7.34
7.30
5.02
0.99
0.98
1.03
4.14
1.11
1.07
2.01
OH
B
O
Cl
Cl
f1 (ppm)

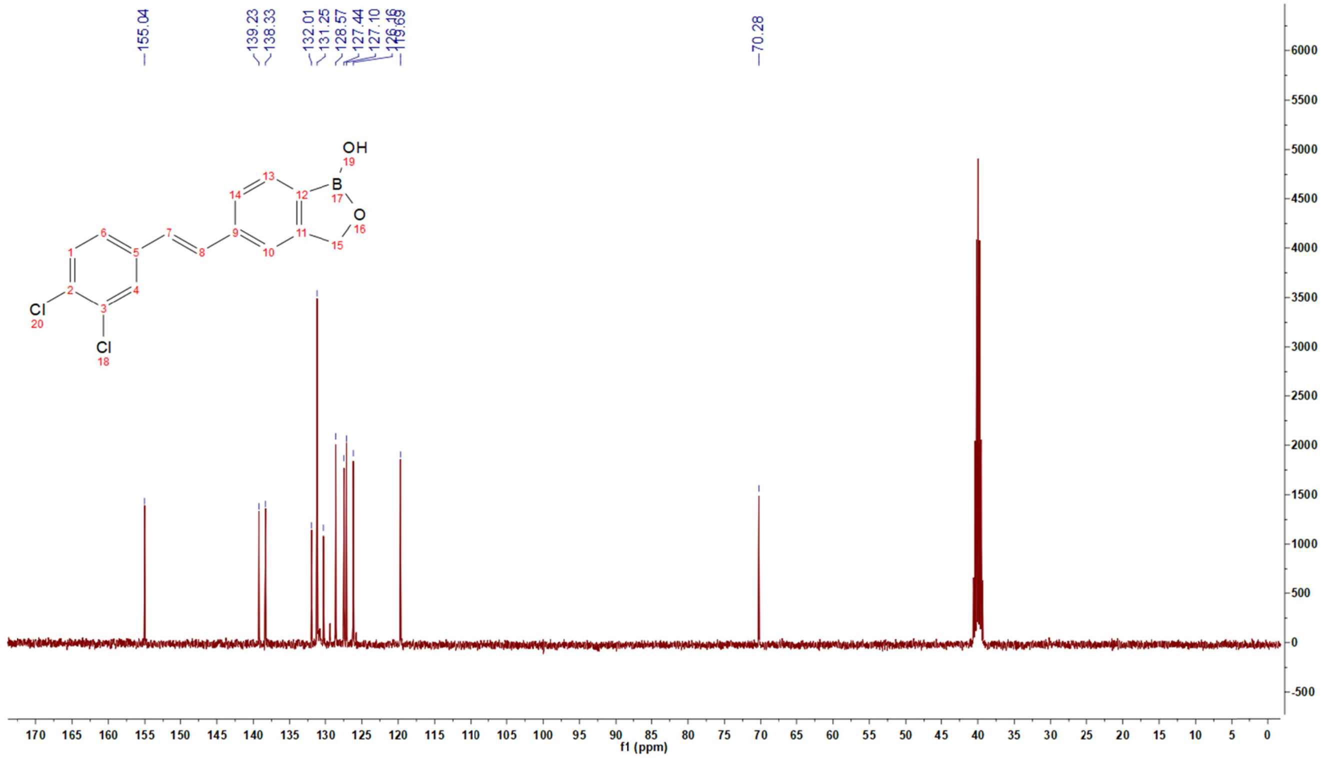
155.04
139.23
138.33
132.01
131.25
128.57
127.44
127.10
126.16
119.69
70.28
OH
B
O
Cl
Cl
f1 (ppm)

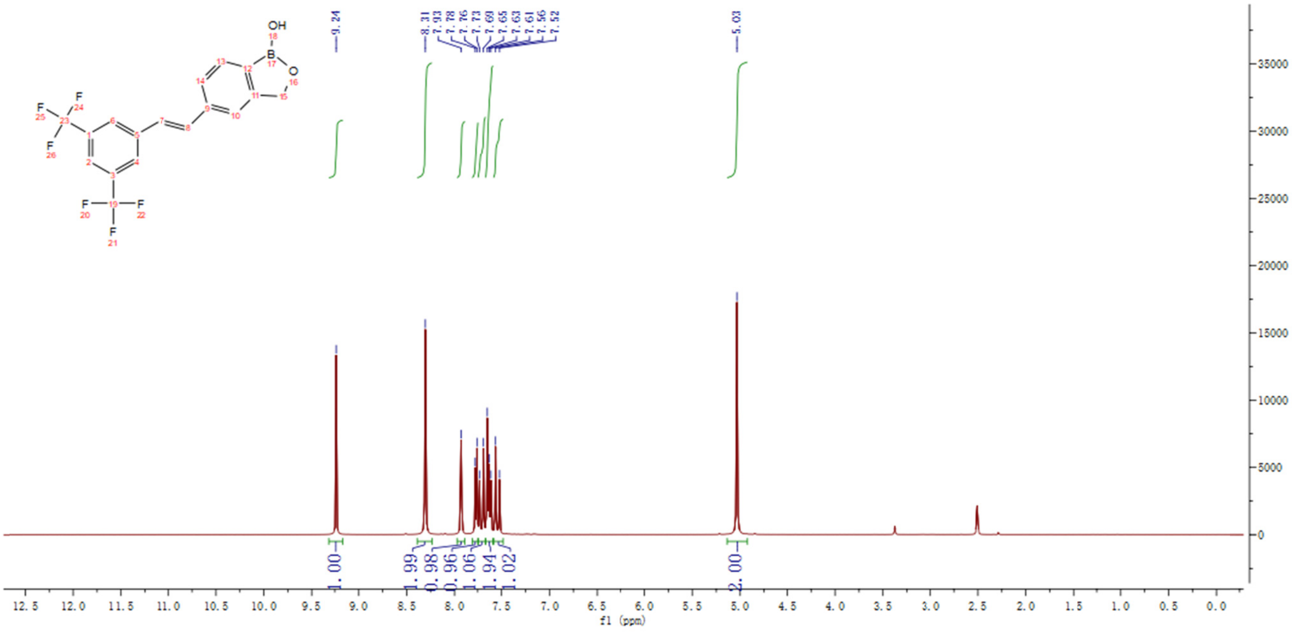

^{13}C NMR (101 MHz, DMSO) δ 155.04, 140.32, 138.97, 133.09, 131.67, 131.34, 131.32, 131.02, 130.69, 127.90, 127.31, 126.94, 126.34, 125.19, 122.48, 120.91, 119.90, 119.77, 70.26.

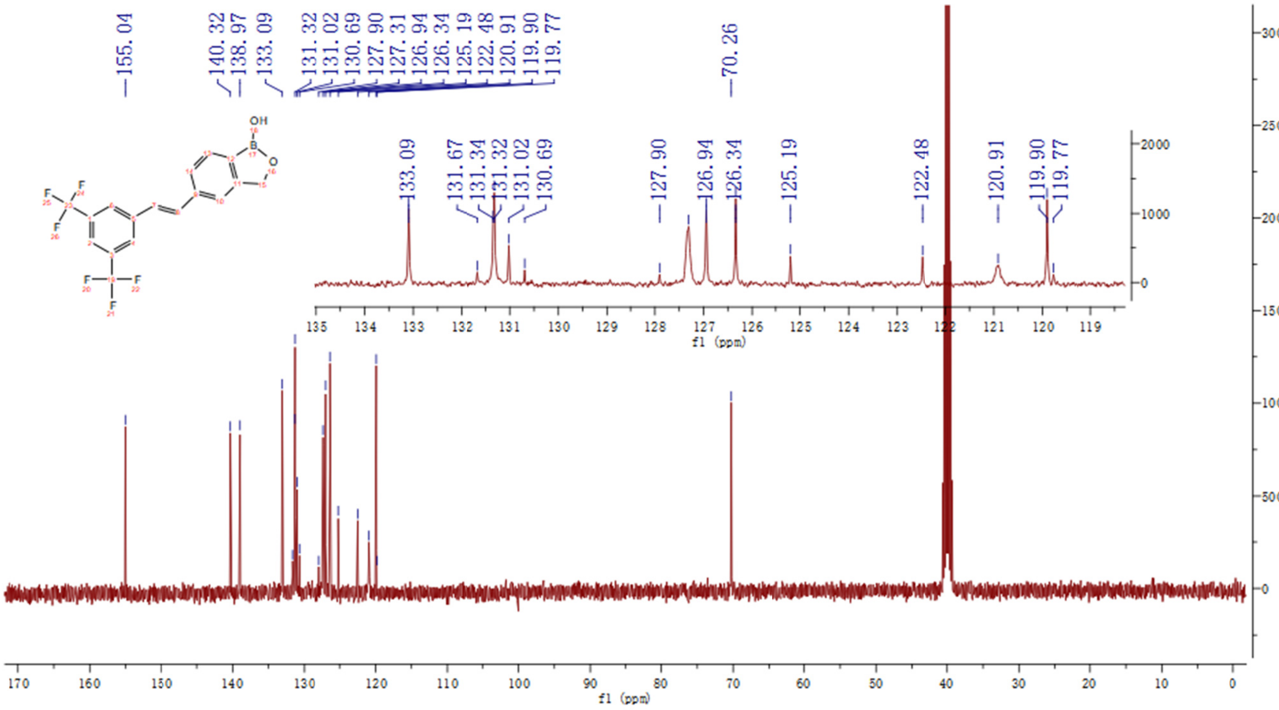

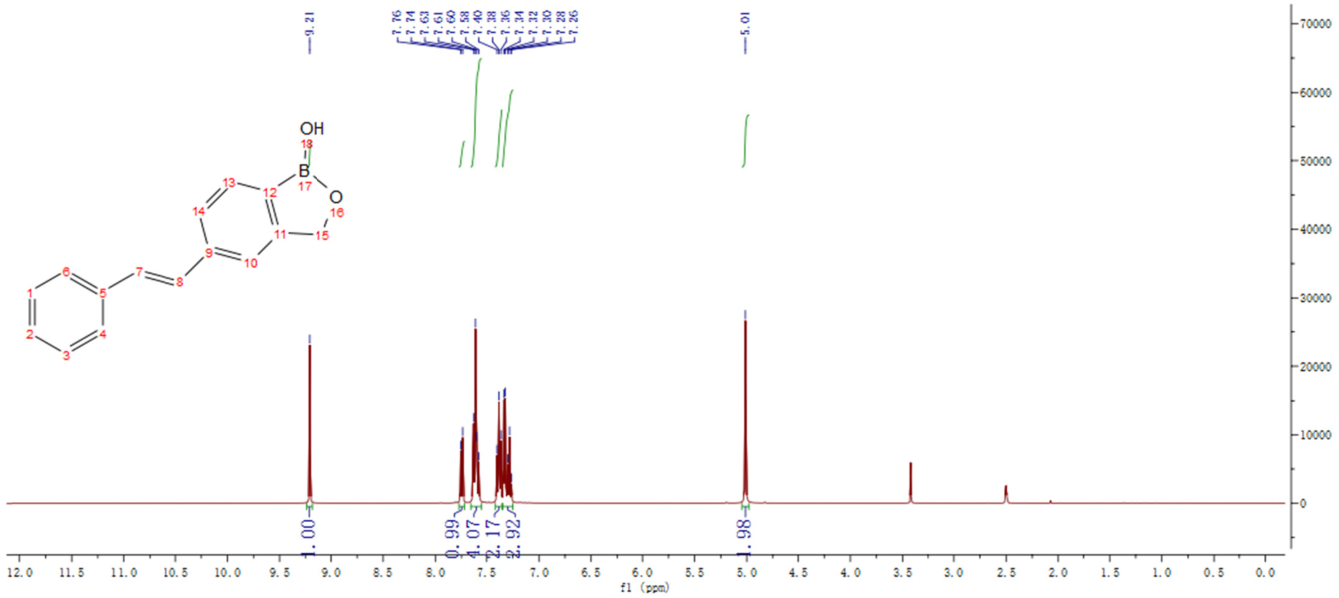
9.21
7.76
7.74
7.63
7.61
7.60
7.58
7.40
7.38
7.36
7.34
7.32
7.30
7.28
7.26
5.01
OH
B
O
1.00
0.99
4.07
2.17
2.92
1.98
f1 (ppm)

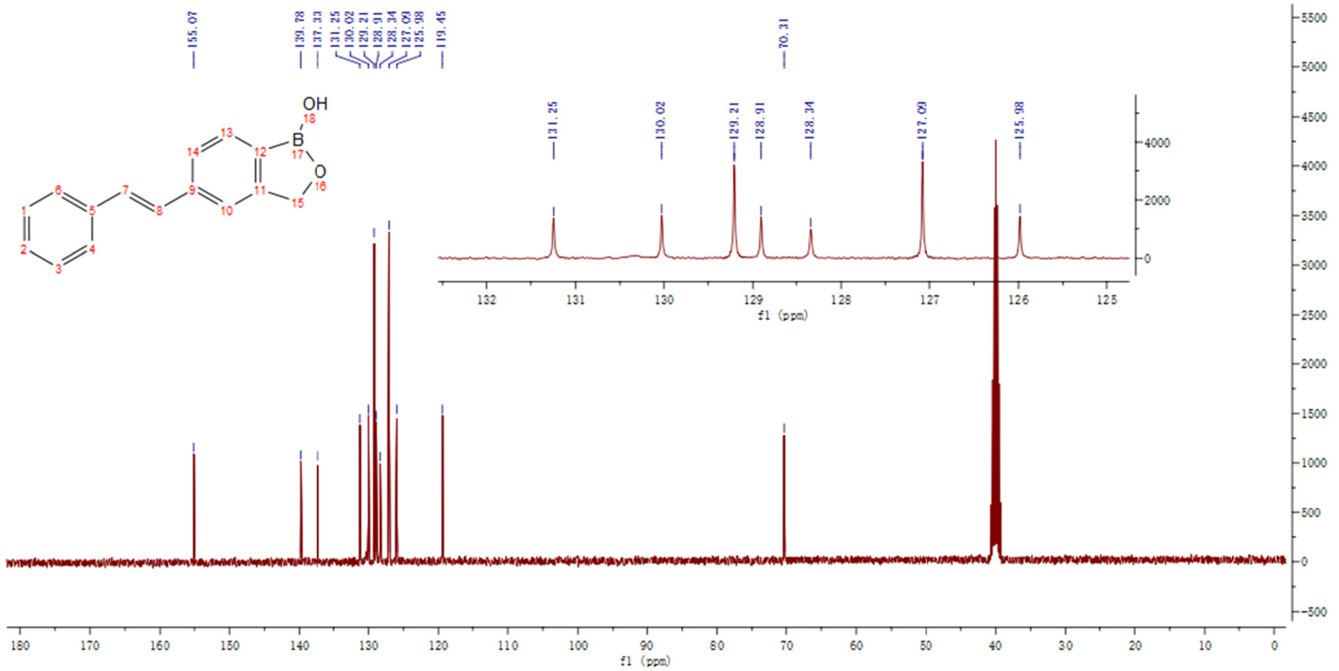
155.07
139.78
137.33
131.25
130.02
129.21
128.91
128.34
127.09
125.98
119.45
70.31
OH
B
O
f1 (ppm)

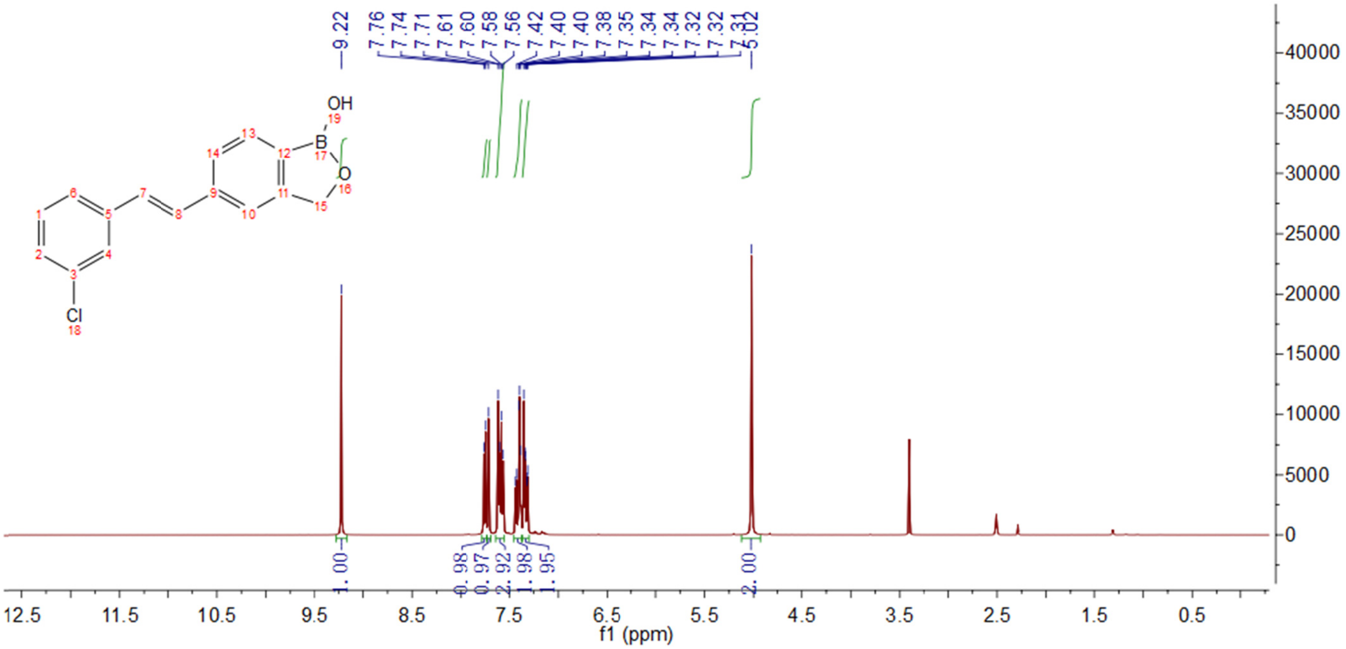

9.22
7.76
7.74
7.71
7.61
7.60
7.58
7.56
7.42
7.40
7.38
7.35
7.34
7.34
7.32
7.31
5.02
OH
B
O
Cl
1.00
0.98
0.97
2.92
1.98
1.95
2.00
f1 (ppm)

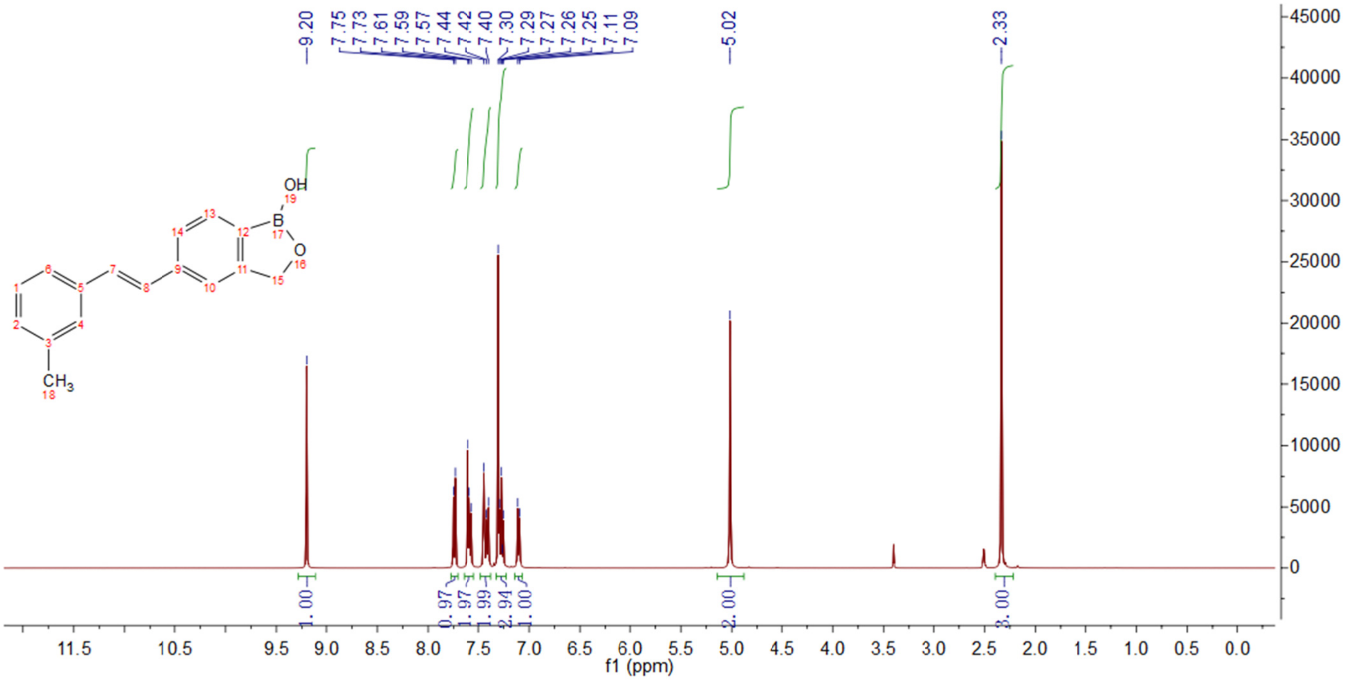

9.20
7.75
7.73
7.61
7.59
7.57
7.44
7.42
7.40
7.30
7.29
7.27
7.26
7.25
7.11
7.09
5.02
2.33
OH
B
O
CH_3
1.00
0.97
1.97
1.99
2.94
1.00
2.00
3.00
f1 (ppm)

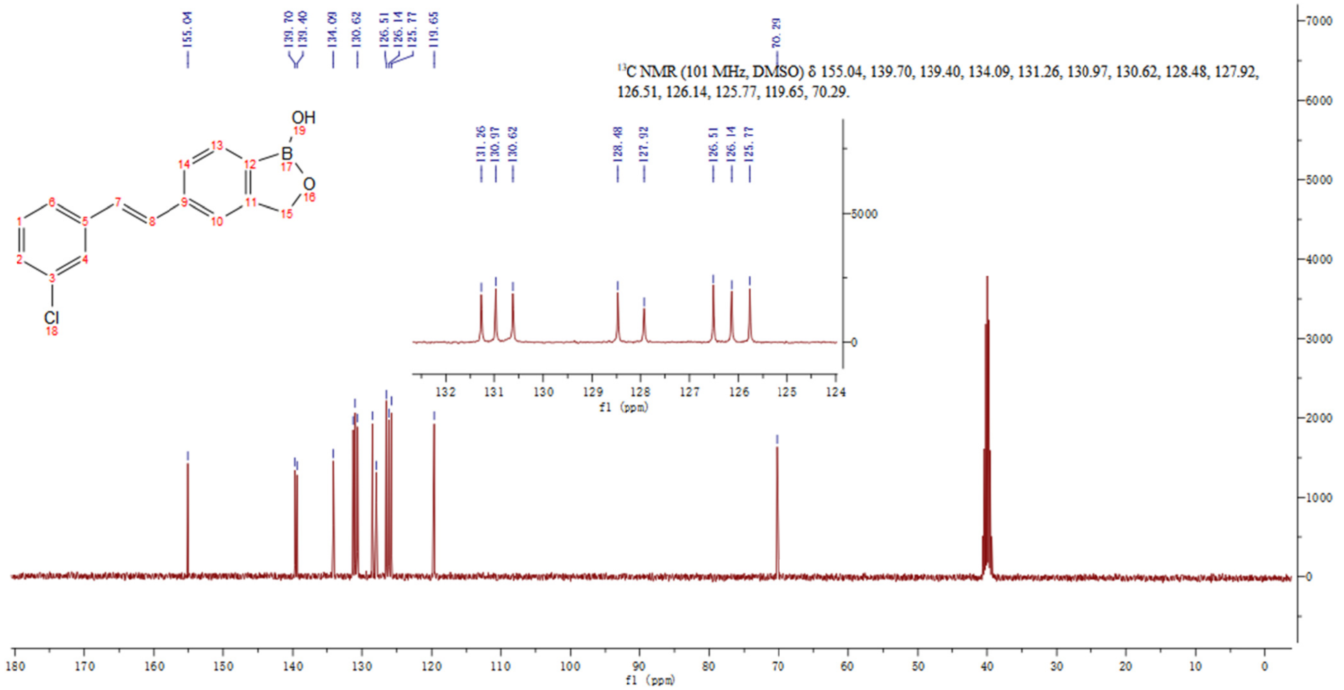

^{13}C NMR (101 MHz, DMSO) δ 155.04, 139.70, 139.40, 134.09, 131.26, 130.97, 130.62, 128.48, 127.92, 126.51, 126.14, 125.77, 119.65, 70.29.
OH
Cl
f1 (ppm)

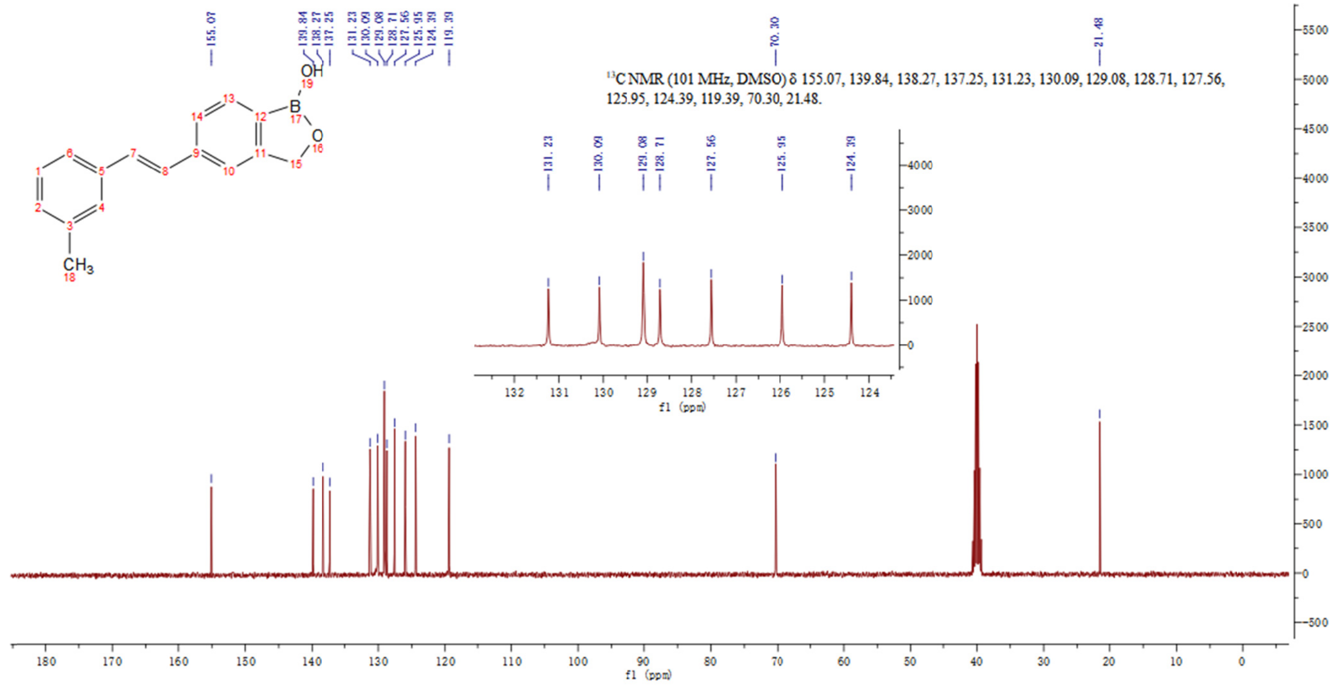

^{13}C NMR (101 MHz, DMSO) δ 155.07, 139.84, 138.27, 137.25, 131.23, 130.09, 129.08, 128.71, 127.56, 125.95, 124.39, 119.39, 70.30, 21.48.
OH
CH_3
f1 (ppm)

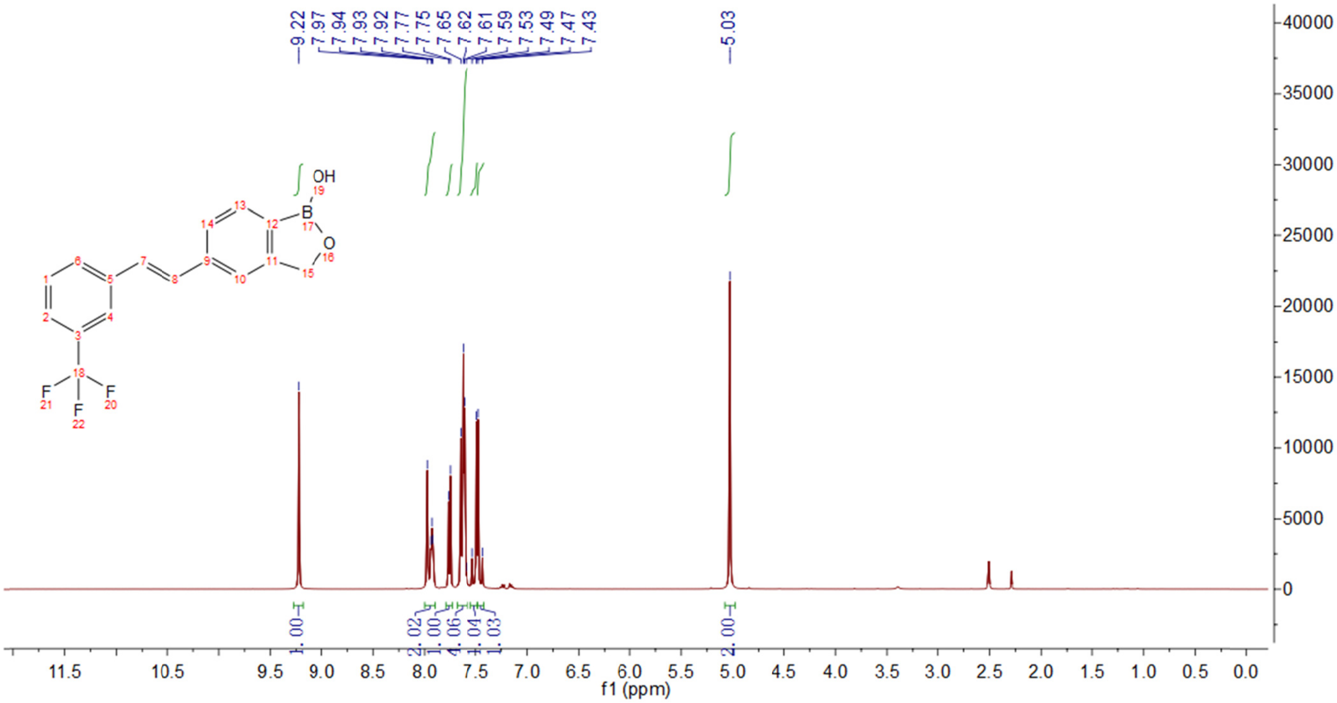

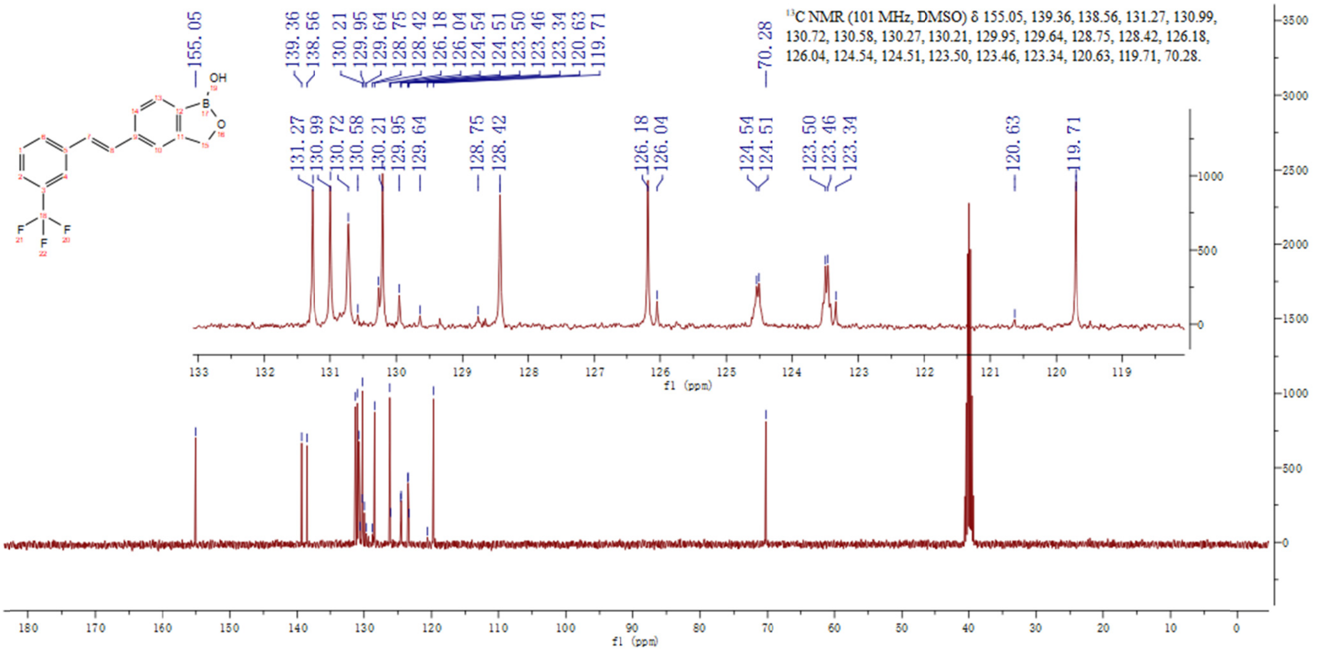

^{13}C NMR (101 MHz, DMSO) δ 155.05, 139.36, 138.56, 131.27, 130.99, 130.72, 130.58, 130.27, 130.21, 129.95, 129.64, 128.75, 128.42, 126.18, 126.04, 124.54, 124.51, 123.50, 123.46, 123.34, 120.63, 119.71, 70.28.
